# Precision Navigation of Hepatic Ischemia–Reperfusion Injury Guided by Lysosomal Viscosity-Activatable NIR-II Fluorescence

**DOI:** 10.1021/jacs.2c03832

**Published:** 2022-07-06

**Authors:** Jihong Liu, Wen Zhang, Chunmiao Zhou, Mengmei Li, Xin Wang, Wei Zhang, Zhenzhen Liu, Luling Wu, Tony D. James, Ping Li, Bo Tang

**Affiliations:** † College of Chemistry, Chemical Engineering and Materials Science, Key Laboratory of Molecular and Nano Probes, Ministry of Education, Collaborative Innovation Center of Functionalized Probes for Chemical Imaging in Universities of Shandong, Institutes of Biomedical Sciences, Shandong Normal University, Jinan 250014, People’s Republic of China; ‡ Department of Chemistry, University of Bath, Bath BA2 7AY, U.K.; § School of Chemistry and Chemical Engineering, Henan Normal University, Xinxiang 453007, People’s Republic of China

## Abstract

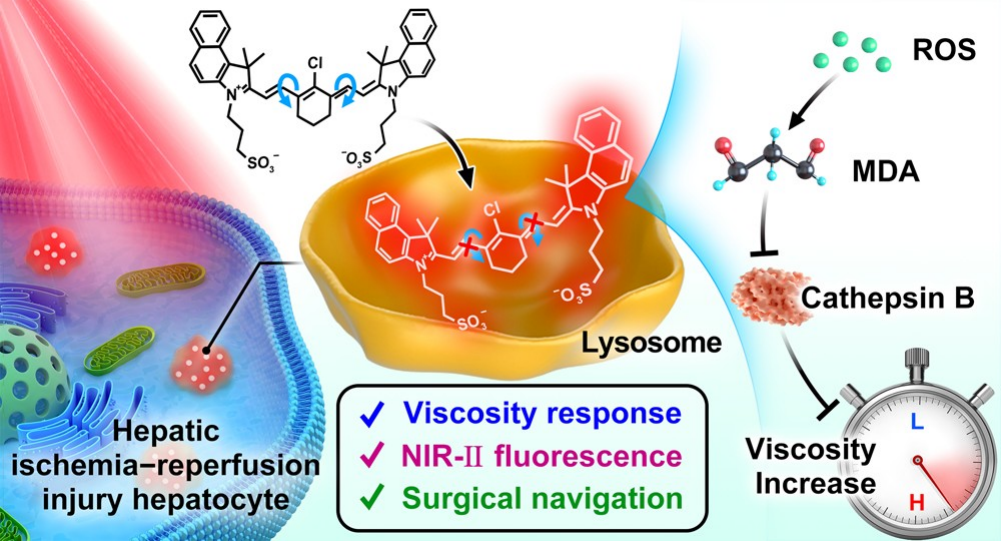

Hepatic ischemia–reperfusion injury (HIRI) is responsible for postoperative liver dysfunction and liver failure. Precise and rapid navigation of HIRI lesions is critical for early warning and timely development of pretreatment plans. Available methods for assaying liver injury fail to provide the exact location of lesions in real time intraoperatively. HIRI is intimately associated with oxidative stress which impairs lysosomal degradative function, leading to significant changes in lysosomal viscosity. Therefore, lysosomal viscosity is a potential biomarker for the precise targeting of HIRI. Hence, we developed a viscosity-activatable second near-infrared window fluorescent probe (NP-V) for the detection of lysosomal viscosity in hepatocytes and mice during HIRI. A reactive oxygen species–malondialdehyde–cathepsin B signaling pathway during HIRI was established. We further conducted high signal-to-background ratio NIR-II fluorescence imaging of HIRI mice. The contour and boundary of liver lesions were delineated, and as such the precise intraoperative resection of the lesion area was implemented. This research demonstrates the potential of NP-V as a dual-functional probe for the elucidation of HIRI pathogenesis and the direct navigation of HIRI lesions in clinical applications.

## Introduction

Hepatic ischemia–reperfusion injury (HIRI) is a common, almost inevitable pathophysiological phenomenon of partial liver resection and transplantation surgery in which interruption in hepatic blood flow is required.^1^ HIRI can lead to 10% of early liver transplant organ failure,^2^ acute and chronic rejection, and liver dysfunction,^3^ which severely affects the prognosis of many patients with hepatic surgery-related diseases. The occurrence of HIRI can also initiate the development of liver inflammation^4,5^ and liver fibrosis.^6,7^ Thus, rapid and accurate targeting of early HIRI lesions can facilitate the timely intervention and treatment and significantly reduce the risks of further deterioration. Traditional clinical diagnosis of liver injury typically includes blood tests, diagnosis by imaging, and liver biopsy. Unfortunately, blood tests using the determination of certain liver enzyme indicator levels cannot visualize in real time and unequivocally determine liver injury.^8^ Imaging modalities including ultrasound, magnetic resonance imaging, and computed tomography, have the limitation of low resolution, leading to an inferior detection rate of microlesions.^9^ Liver biopsy is recognized as the gold standard for clinical diagnosis of liver injury, but it is only confirmed postoperatively, furthermore, both high variabilities among observers and sampling errors in samples result in poor evaluation accuracy.^10^ Hence, the development of an in situ HIRI detection method that enables intraoperative visualization in real time, high resolution, and provides the precise location of the injury site is urgently required.

Lysosomes are degradation sites for damaged proteins and organelles in cells, to which antiquated organelles or macromolecules are transported for degradation and recycling. However, in the event of lysosomal dysfunction, macromolecules are unable to decompose and accumulate in excess in the lysosomes, ultimately leading to significant changes in lysosomal viscosity.^11,12^ Increasing evidence suggests that lysosomal degradative functions are closely regulated by oxidative stress.^13^ In particular, oxidative stress in the liver could damage lysosomal degradation, eventually resulting in liver injury.^14,15^ Since the pathogenesis of HIRI is intimately associated with oxidative stress disorder,^16^ lysosomal viscosity can be considered as a key parameter of HIRI to effectively distinguish HIRI tissues from normal liver tissues. Moreover, research has suggested that inflammation or necrosis may result in edema, which eventually causes liver viscosity changes via enhancing the internal pressure in the liver.^17^ Therefore, the development of effective tools for detecting lysosomal viscosity, enabling visualization in real time and accurate localization of HIRI injury sites, is a challenging and essential requirement.

Due to the advantages of excellent spatial resolution, high sensitivity, and good selectivity, fluorescence imaging has great potential for the real-time monitoring of intracellular bioactive small molecules^18−23^ and key parameters related to the cell microenvironment, including pH,^24,25^ polarity,^26^ and viscosity.^27,28^ In particular, second near-infrared window (NIR-II, 1,000–1,700 nm) fluorescence imaging has attracted significant interest since it integrates centimeter depth tissue penetration, ultrahigh micron resolution at millimeter depth, and ultrahigh signal-to-background ratio (SBR) imaging.^29,30^ To date, the potential application of the NIR-II fluorescence imaging window for the diagnosis of diseases and image-guided surgery has been extensively explored. NIR-II fluorescent probes can function as a surgeon’s third eye, aiding the surgeon in the identification and removal of all lesions, which is particularly important for preoperative diagnosis and intraoperative navigation.^31^ For example, Zhang et al. developed peptide targeting and DNA-modified nanoparticles, where ovarian metastases at ≤1 mm could be identified and resected via *in vivo* assembly under image guidance.^32^ Tian et al. used indocyanine green (ICG) to assist the resection of primary and metastatic liver tumors in 23 patients guided by the first near-infrared window (NIR-I) and NIR-II fluorescence imaging, facilitating the clinical transformation of NIR-II fluorescence imaging.^9^ Small-molecule-based probes have exhibited excellent performance, such as ease of structural modification,^33^ rapid excretion capacity,^34^ and extremely low toxicities in living organisms.^35^ This encouraged us to develop a viscosity-activatable small-molecule NIR-II fluorescent probe to allow the nondestructive localization of HIRI lesions *in vivo* and enable the precise surgical resection of the lesions.

Herein, we fabricated a NIR-II fluorescent probe (NP-V) with a viscosity-specific response and performed imaging and surgical resection of liver lesions with HIRI mice. Facilitated by NP-V, we determined that reactive oxygen species (ROS)–malondialdehyde (MDA)–cathepsin B mediated the molecular mechanism of lysosomal viscosity variation in HIRI and determined that lysosomal viscosity could be an ideal biomarker for the precise navigation of HIRI lesion sites. Importantly, high resolution and superior SBR NIR-II fluorescence facilitated HIRI lesions’ surgical delineation. Histopathology examinations confirmed that the pathological tissues in the liver of HIRI mice could be precisely excised under NIR-II fluorescence guidance.

## Results and Discussion

### Design and Synthesis of NP-V

Recently, many fluorescent probes have been developed for the detection of viscosity. However, most of their emissions are in the visible to NIR-I window (400–900 nm), which hinders their biological and biomedical application.^36^ Hence, we set out to design imaging tools that respond to viscosity in the NIR-II window. Rational design strategies for NIR-II polymethine dyes include extended polymethine chains, large π-conjugated structures, as well as increased rigidity of both the polymethine chain and terminal groups.^37^ In particular, ICG exhibits a low quantum yield and is highly susceptible to photobleaching.^38^ As such, increasing the structural rigidity can enhance the quantum yield and photostability.^37,39^ For IR-783, attaching benzene rings to the terminal groups improves the bathochromic shift of the absorption and emission wavelengths, such as FD-1080.^37^ Therefore, the disadvantages of using ICG or IR-783 can be resolved using a combination of the two modifications.

**Figure 1:**
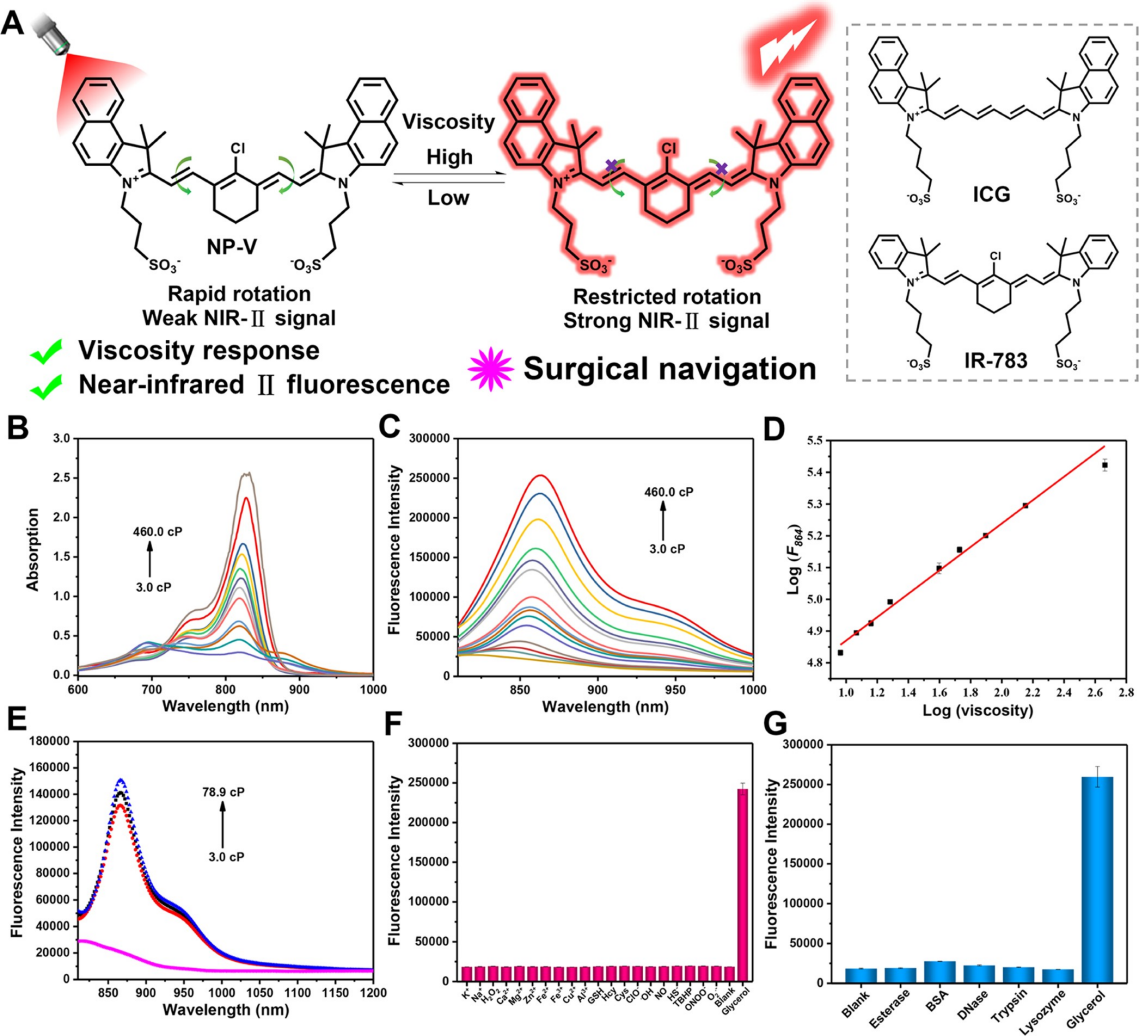
Structure and fluorescence properties of viscosity-responsive NP-V. (A) Luminescence mechanism for viscosity and the chemical structures of ICG and IR-783. (B) Absorption spectra of NP-V (5 μM) in a water–glycerol system with different viscosities. (C) Fluorescence spectra of NP-V (5 μM) in water/glycerol mixtures with different viscosities in the emission window of 810–1000 nm. (D) Linear relationship between log *F*_864_ and log η. (E) NIR-II fluorescence responses of NP-V (5 μM) to various viscosities at emission wavelengths from 810 to 1200 nm. (F) Fluorescence response of NP-V (5 μM) to various ROS, RNS, and metal ions (5 mM K^+^, 5 mM Na^+^, 1 mM H_2_O_2_, 200 μM Ca^2+^, 200 μM Mg^2+^, 200 μM Zn^2+^, 200 μM Fe^2+^, 200 μM Fe^3+^, 200 μM Cu^2+^, 200 μM Al^3+^, 100 μM GSH, 100 μM Hcy, 200 μM Cys, 10 μM ClO^–^, 10 μM ^•^OH, 10 μM NO, 10 μM HS^–^, 10 μM TBHP, 10 μM ONOO^–^, 10 μM O_2_^•–^, blank, and 95% glycerol). (G) Fluorescence response of NP-V (5 μM) to abundant enzymes *in vivo* (blank, 1.0 kU/L esterase, 2 μM BSA, 5.0 kU/L DNase, 300 mg/L trypsin, 15 mg/L lysozyme, and 95% glycerol). All fluorescence spectra were acquired at λ_ex/em_ = 808/864 nm.

Our design strategy for NP-V is given in Figure 1A. First, NP-V integrates the structural superiority of ICG and IR-783, and further facilitates the red-shifted fluorescence emission by adding multiple aromatic rings and the installation of a rigid cyclohexenyl. As such the large π-conjugated system with enhanced rigidity extended the NIR emission wavelength of NP-V. Furthermore, by introducing a rigid cyclohexenyl substituent in the central position, the photostability and quantum yield of cyanine dyes can be significantly boosted via decreased nonradiative transitions,^37^ significantly improving the clinical translational potential of NP-V. Second, benzindoles were connected to the cyclohexene by flexible conjugated bonds, rendering NP-V highly sensitive to viscosity changes. Third, sulfonate groups enhance the water solubility and biocompatibility of NP-V, facilitating the rapid clearance of NP-V *in vivo*.^40^ NP-V was synthesized via the Vilsmeier–Haack reaction^41^ and its chemical structure was determined using HRMS, ^1^H NMR, and ^13^C NMR (Figures S21, S22, and S23).

### Optical Properties of NP-V *In Vitro*

To characterize the optical properties of NP-V, we first investigated the spectral response of NP-V to viscosity in a water–glycerol system.^42−44^ As shown in Figure 1B, NP-V exhibited a weak absorption peak at about 694 nm in water with a low viscosity. As the viscosity of the solvent medium was increased, the absorption peak of NP-V at 694 nm almost disappeared, accompanied by an obviously elevated absorption peak at 820 nm. The red-shifted absorbance at 820 nm can be attributed to the increased conjugation of NP-V, due to the formation of a more planar configuration in higher-viscosity solvents. Notably, under 808 nm excitation, NP-V displayed a 13-fold fluorescence enhancement at 864 nm as the medium viscosity increased from 3.0 cp to 460.0 cp (Figure 1C). Specifically, the fluorescence intensity of NP-V at 864 nm (log *F*_864_) exhibited a good linear relationship with the viscosity of the medium (log η, 9.2 cp-460.0 cp). The linear equation was log *F*_864_ = 0.3685 log η + 4.5016 with a linear coefficient of 0.997 (calculated by Förster–Hoffmann equation,^45^Figure 1D). As shown in Figure S1, the fluorescence quantum yield (Φ_f_) of NP-V in water was calculated as 0.037, but in a maximally viscous solution (100% glycerol), the fluorescence quantum yield of NP-V increased to 0.34, which was 2.6-fold higher than that of ICG in DMSO (Φ_f_ = 0.13). In a low viscous solvent, the rotatable single bonds and excited-state C=C double bonds of NP-V can result in a nonplanar structure of NP-V and energy loss through nonradiative pathways, this ultimately leads to fluorescence quenching. However, the constraints of high viscosity solvents made rotatable single bonds and excited-state C=C double bonds rigid in NP-V, causing restricted intramolecular rotation (RIR), which results in fluorescence enhancement. Conspicuously, as the medium viscosity increased from 3.0 to 78.9 cp, NP-V exhibited bright NIR-II fluorescence in the 900–1050 nm emission window, and the tail of the emission spectrum extended to 1200 nm (Figure 1E), indicating a suitable viscosity response for NP-V in the NIR-II region. The above results confirmed the excellent sensitivity of NP-V toward viscosity and robust NIR-II fluorescence emission.

To interrogate the specificity of NP-V toward viscosity, we examined its selectivity by recording reactions with various ROS, reactive nitrogen species (RNS), and proteins under simulated physiological conditions. Encouragingly, only an increase in viscosity resulted in a significant increase of the fluorescence signal at 864 nm without any interference from other highly active ROS, RNS, metal ions, or abundant proteins *in vivo* (Figure 1F,G), demonstrating that NP-V had high specificity for viscosity. Importantly, we also observed that environmental polarity changes from different solvents had no effect on the fluorescence intensity of NP-V (Figure S2). Furthermore, the fluorescence intensity of NP-V at 864 nm hardly changed in PBS buffer of different pH values. However, in the presence of glycerol, NP-V exhibited an obvious enhancement of red fluorescence over a wide pH range (pH 5.0–9.0) without significant differences, indicating that NP-V exhibited excellent pH stability (Figure S3). Moreover, NP-V displayed good photostability in media with different viscosities (Figure S4). The photobleaching properties of NP-V in water were examined under continuous irradiation with an 808 nm laser (900 mW/cm^2^) for 760 s. In contrast with ICG, NP-V did not show any significant photobleaching even after continuous exposure to irradiation from the 808 nm laser for 300 s, indicating the high resistance of NP-V to photobleaching (Figure S5). Given that an image-guided probe with excellent biocompatibility is in high demand, we then evaluated the cytotoxicity of NP-V. From cytotoxicity assays, the IC_50_ value of NP-V was 1.33 mM (Figure S6). As such, NP-V exhibits several advantages such as high sensitivity, good selectivity, wide pH tolerance, and low cytotoxicity, which make it a promising candidate for viscosity detection in complex biological systems.

### Fluorescence Imaging of Viscosity Variations at the Subcellular Level

**Figure 2:**
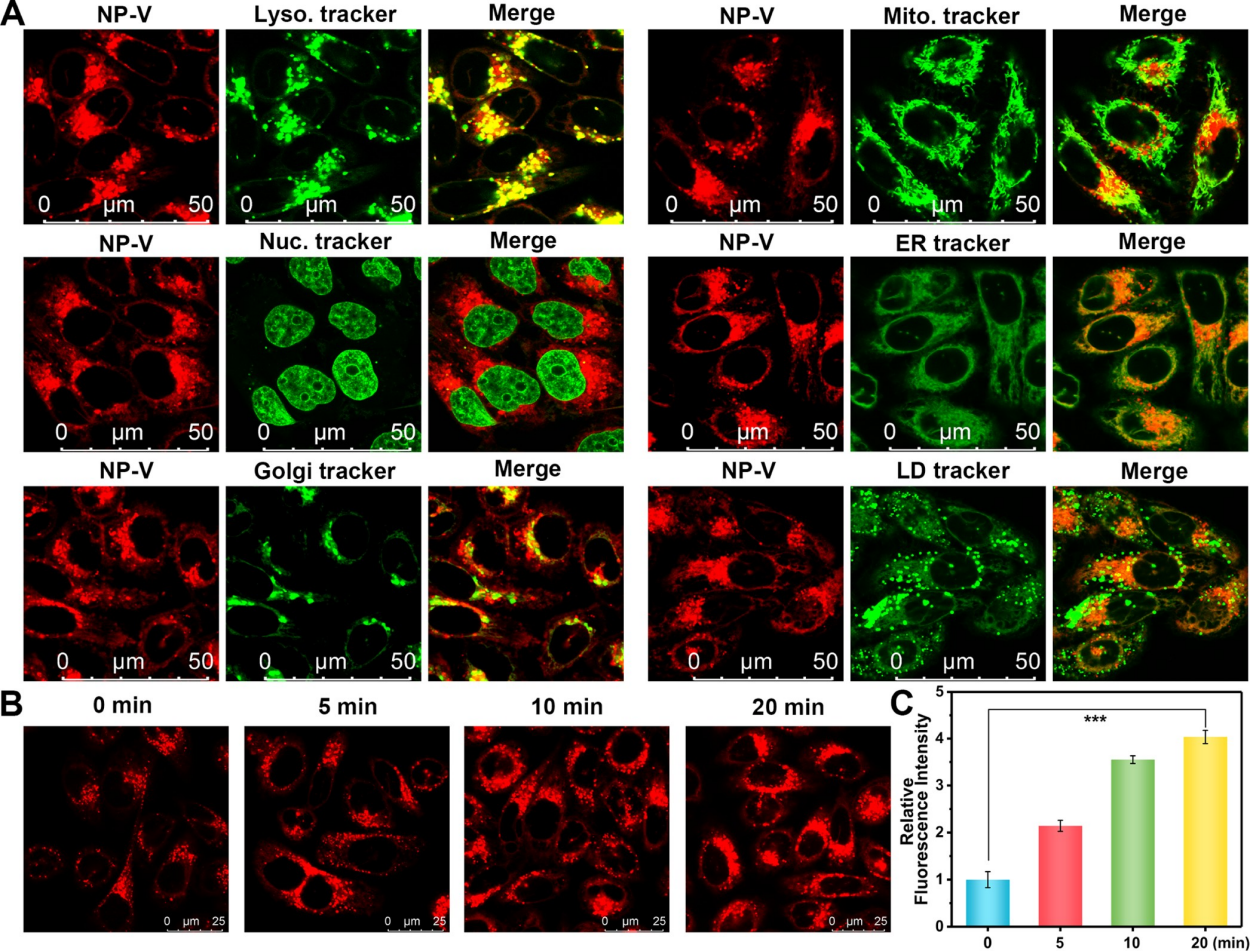
Subcellular localization and fluorescence imaging of viscosity changes in hepatocytes by NP-V. (A) Colocalization images of hepatocytes coincubated with NP-V for the red channel (20 μM, Ex = 633 nm, collected from 640–820 nm) and the corresponding organelle targeting dyes for the green channel (LysoTracker Green, 75 nM, Ex = 488 nm, collected from 490–550 nm; MitoTracker Green, 60 nM, Ex = 488 nm, collected from 490–550 nm; Hoechst 33342, 1 μg/mL, Ex = 405 nm, collected from 423–483 nm; ER-Tracker Green, 100 nM, Ex = 488 nm, collected from 490–529 nm; Golgi-Tracker Green, 100 nM, Ex = 488 nm, collected from 490–529 nm; BODIPY 493/503, 1 μg/mL, Ex = 488 nm, collected from 490–550 nm). (B) Detection of lysosomal viscosity dynamics by NP-V (20 μM) in 5 μM dexamethasone-pretreated (0, 5, 10, 20 min) hepatocytes. Ex = 633 nm, collected from 640–820 nm. (C) Relative fluorescence intensity output of (B). The fluorescence intensity of the dexamethasone-pretreated 0 min group was defined as 1. The data are expressed as the mean ± SD. ***P < 0.001. Concordant results were obtained from three independent experiments.

Motivated by the photophysical properties of NP-V, we then investigated the applicability for fluorescence imaging of viscosity changes at the subcellular level. Here, we chose an excitation wavelength of 633 nm for intracellular imaging, as it is the longest available NIR excitation wavelength for a confocal high-resolution fluorescence microscope. Under 633 nm excitation, the fluorescence enhancement behavior of NP-V toward the increased viscosity in water/glycerol mixtures was confirmed (Figure S7). The intracellular photostability of NP-V was evaluated under continuous irradiation using a 633 nm laser (intensity 32.5%) of the confocal high-resolution fluorescence microscope for 460 s. Notably, no obvious red fluorescence intensity variation of NP-V was detected under irradiation with a 633 nm laser for 200 s, whereas a significant decrease in the red fluorescence intensity was found in HL-7702 cells stained with ICG. These results suggest that NP-V exhibits intracellular photostability without suffering severe photobleaching (Figure S8). To delineate the subcellular localization of NP-V in the fluorescence imaging, we costained hepatocytes with NP-V and a series of commercial organelle dyes, such as Lysosome Tracker Green, Mitochondria Tracker Green, Hoechst 33342 Endoplasmic Reticulum Tracker Green, Golgi apparatus Tracker Green, and BODIPY 493/503,^46−48^ and exposed them to continuous colocalization imaging. As illustrated in Figure 2A, NP-V accumulated in hepatocyte lysosomes and presented red dot fluorescence, which was well fused with lysosomal green fluorescence with a Pearson’s colocalization coefficient of 0.91. However, Pearson’s colocalization coefficients with mitochondria, endoplasmic reticulum, Golgi apparatus, and lipid droplet markers were 0.37, 0.40, 0.21, and 0.39, respectively. The specific targeting of NP-V to lysosomes can be explained based on two aspects. First, NP-V molecules tended to bind with serum proteins in cell culture medium and form protein-sized “nanoparticles”,^49^ which was confirmed by dynamic light scattering (DLS) and zeta potential experiments. After the addition of NP-V, the average size of nanoparticles in the cell culture medium was increased, while the zeta potential was reduced (Figures S9 and S10). The as-formed nanoparticles enter the cell through endocytosis, passing through a series of processes, such as the early endosomes and the late endosomes, and eventually arrive at the lysosomes.^50^ Second, lysosomes are highly viscous cell organelles (47–190 cp at 25 °C),^51,52^ as a consequence, NP-V could be specifically activated by the highly viscous lysosomal microenvironment. Therefore, both endocytosis and the highly viscous environment in lysosomes enabled NP-V to be detected in lysosomes. To determine whether the intracellular fluorescence response of NP-V to viscosity was affected by serum protein, we evaluated the fluorescence response of NP-V to viscosity in a cell culture media environment. NP-V displayed a good fluorescence response to viscosity in cell culture media (Figures S11 and S12). Overall, NP-V can target the lysosomes of hepatocytes specifically and retain a stable fluorescence response toward viscosity in cell culture media.

It has been reported that the local viscosity of the lysosome increases after treatment with ionophores (dexamethasone, *etc*.).^12,51^ Therefore, the feasibility of using NP-V to detect dynamic changes of viscosity in lysosomes under dexamethasone treatment was evaluated. HL-7702 cells were incubated with dexamethasone, followed by staining with NP-V. As shown in Figures 2B,C, the red fluorescence in lysosomes was remarkably enhanced after stimulation with dexamethasone. Compared with the control group, hepatocytes that were pretreated with 5 μM dexamethasone for 20 min exhibited a 4.0-fold fluorescence enhancement. Interestingly, a clear dot signal emerged in hepatocytes after being exposed to dexamethasone, indicating that dexamethasone could increase the lysosomal viscosity without destroying the integrity of the lysosome. These observations indicate that NP-V can image the dynamic changes of lysosomal viscosity in real time under the stimulation of dexamethasone.

### Real-Time Imaging for the Detection of Lysosomal Viscosity Variations in Hepatocytes during HIRI

**Figure 3:**
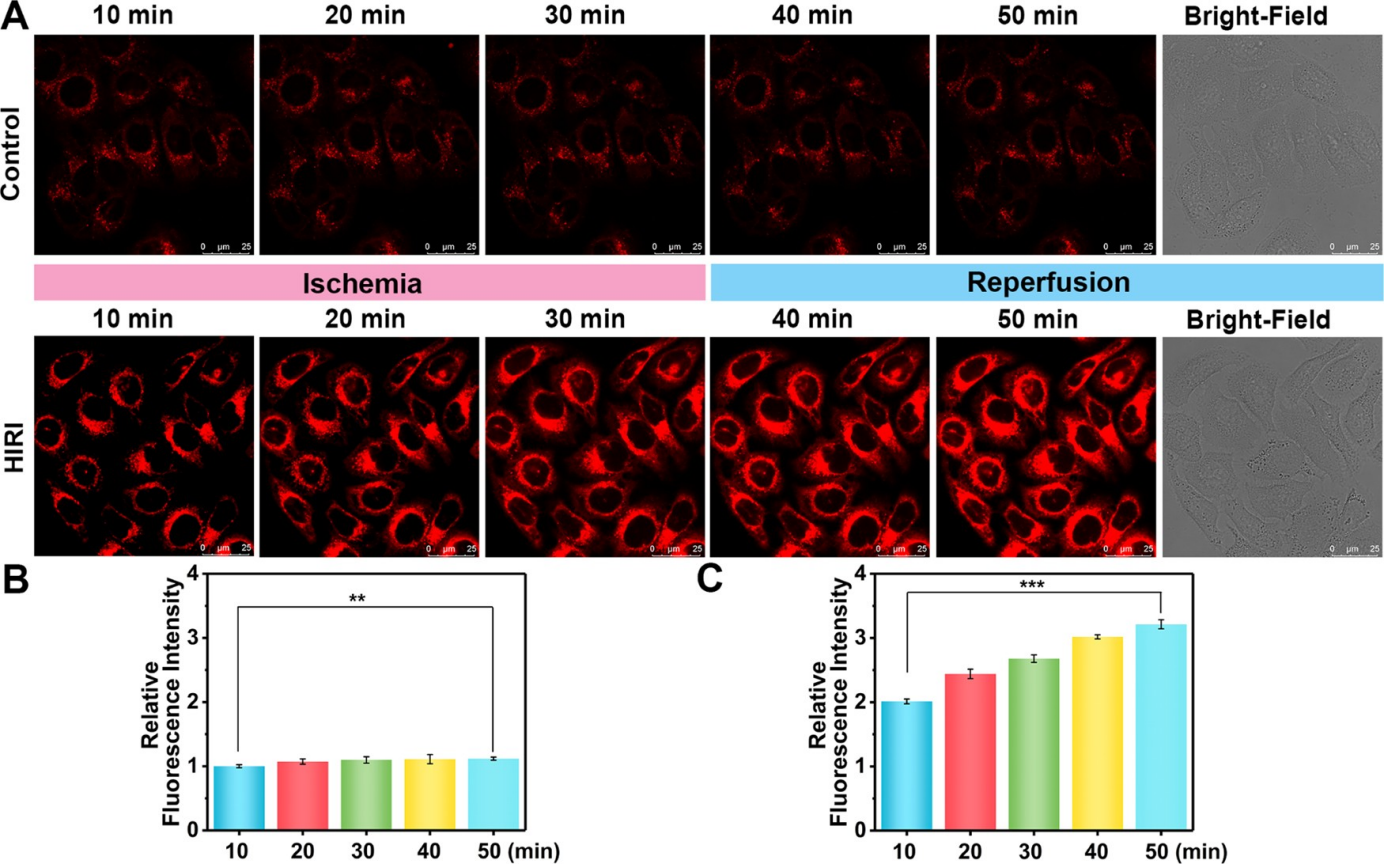
Real-time fluorescence imaging of lysosomal viscosity in hepatocytes during the HIRI process. (A) Fluorescence images of lysosomal viscosity in the 20 μM NP-V-loaded control group and HIRI group at 10, 20, 30, 40, and 50 min. Ex = 633 nm, collected from 640–820 nm. (B) Relative fluorescence intensity output of the control group. (C) Relative fluorescence intensity output of the HIRI group. The fluorescence intensity of the control group was defined as 1 at 10 min. The data are expressed as the mean ± SD. **P < 0.01 and ***P < 0.001. Concordant results were obtained from three independent experiments.

Encouraged by the excellent intracellular imaging capability of NP-V, the dynamic variations of lysosomal viscosity during the process of HIRI were then explored. We first established a HIRI model in hepatocytes following a previously reported method using oxygen–glucose–serum deprivation for 0–30 min and subsequent reperfusion for 31–50 min.^53,54^ Both the control group and the HIRI group were incubated with NP-V before imaging. As shown in Figure 3, the red fluorescence of the control group stained with NP-V was very weak and no obvious changes were observed, while the HIRI group exhibited a time-dependent red fluorescence signal enhancement. It is particularly worth noting that HIRI cells undergoing ischemia for 30 min and reperfusion for 20 min displayed a 2.9-fold fluorescence signal enhancement in comparison to the control group, indicating that lysosomal viscosity in HIRI hepatocytes was significantly higher than that in normal hepatocytes. Our findings are consistent with a previous report that hepatic oxidative stress could induce lysosomal degradation dysfunction and eventually lead to hepatocellular damage;^14,15^ moreover, lysosomal microenvironmental viscosity could respond sensitively to biomacromolecule accumulation in the lysosomes.^11^ As such, these results suggest that lysosomal viscosity can be regarded as a key parameter of HIRI to effectively distinguish normal liver cells from HIRI cells, and NP-V can realize real-time visual detection of viscosity in HIRI.

### Potential Signaling Pathway for Lysosome-Mediated Viscosity Changes during HIRI

The above experimental results indicated that lysosomal viscosity could serve as an ideal reporter for HIRI evaluation, but the molecular mechanism is unclear. Therefore, we investigated potential signaling pathways for lysosome-mediated viscosity changes in HIRI.

### ROS Accumulation within the Lysosome Induces Lysosomal Viscosity Increase during HIRI

**Figure 4:**
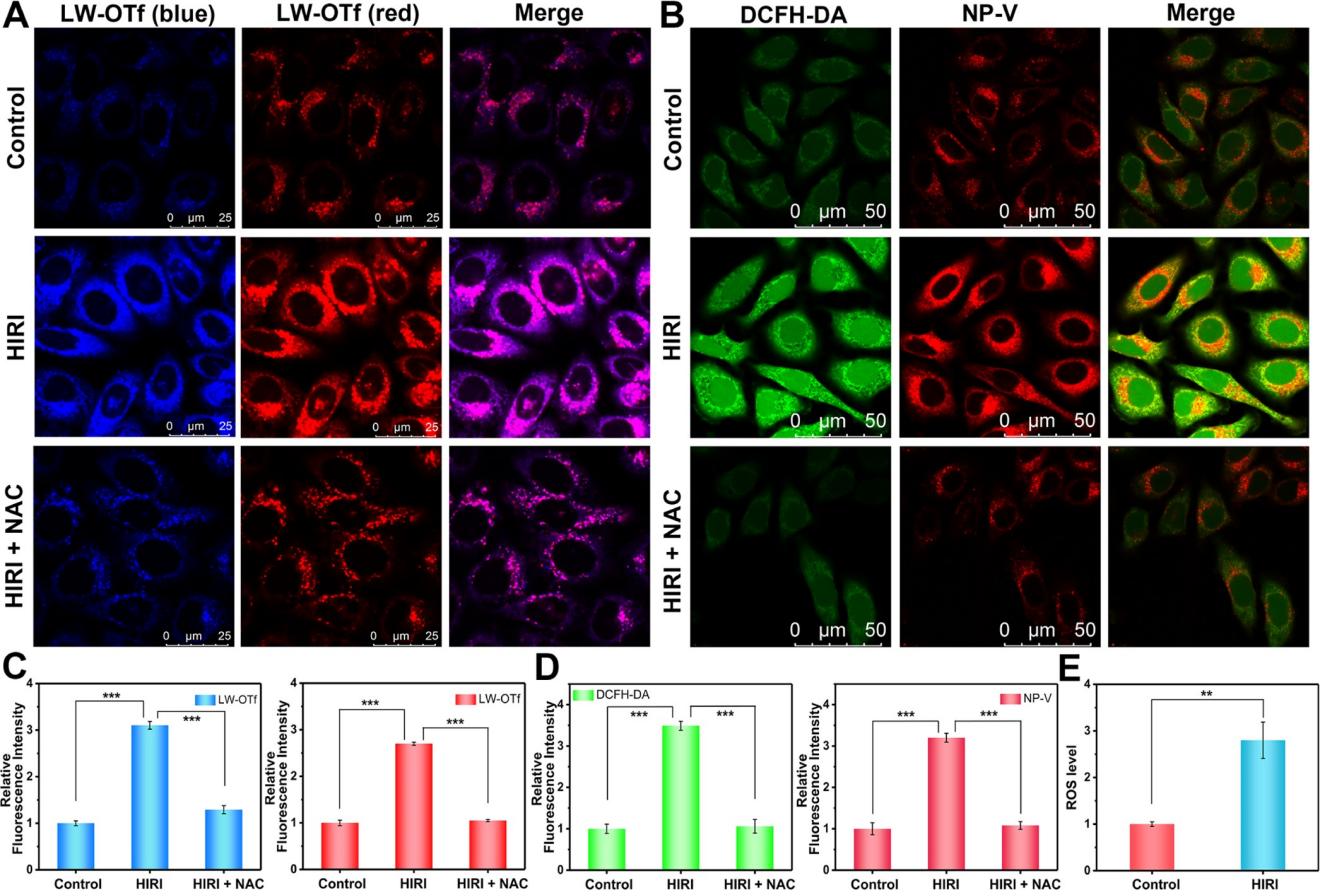
Real-time fluorescence imaging of ONOO^–^, O_2_^•–^ and viscosity in hepatocyte lysosomes during HIRI. (A) Simultaneous fluorescence imaging of ONOO^–^ (blue channel, Ex = 405 nm, collected from 410–500 nm) and O_2_^•–^ (red channel, Ex = 633 nm, collected from 680–780 nm) in hepatocyte lysosomes by LW-OTf. Control, HIRI, and HIRI + NAC groups were stained with 2.4 μM LW-OTf for the blue and red channels. (B) Simultaneous fluorescence imaging of intracellular ROS (green channel, Ex = 488 nm, collected from 490–540 nm) and lysosomal viscosity (red channel, Ex = 633 nm, collected from 640–800 nm) in hepatocytes by DCFH-DA and NP-V. Control, HIRI, and HIRI + NAC groups were stained with 10 μM DCFH-DA for the green channel and 20 μM NP-V for the red channel. (C, D) Relative fluorescence intensity output of (A), (B), respectively. The fluorescence intensity of the control group was defined as 1. (E) Relative ROS level assays in lysosomes of the control group and HIRI group. Data are expressed as the mean ± SD. **P < 0.01 and ***P < 0.001. Concordant results were obtained from three independent experiments.

Evidence suggests that ROS is intimately associated with cell microenvironmental viscosity. On the one hand, viscosity variation influences intracellular basic processes including signal transmission and the efficiency of bimolecular processes due to the diffusion of short-lived intermediates, such as ROS under oxidative stress.^55^ On the other hand, ROS affects the changes in viscosity by oxidizing intracellular components.^56^ First, ROS fluctuations in the lysosomes of the control and HIRI groups were examined. To perform the evaluation, we used LW-OTf which is a previously reported probe for superoxide anion (O_2_^•–^) and peroxynitrite (ONOO^–^), which are two primary ROS and RNS in lysosomes.^48^ The LW-OTf, which exhibits lysosome-specific targeting capacity, enabled real-time imaging and simultaneous discrimination of O_2_^•–^ and ONOO^–^ in two well-separated fluorescence channels. As illustrated in Figure 4A, the control group hepatocytes exhibited weak dot fluorescence in the red and blue fluorescence channels. In comparison to the control group, HIRI group hepatocytes exhibited a 2.7-fold enhancement in O_2_^•–^-related red fluorescence and a 3.1-fold increase in ONOO^–^-associated blue fluorescence, indicating that excessive O_2_^•–^ and ONOO^–^ were produced in the lysosomes of hepatocytes during HIRI (Figure 4C). HIRI hepatocytes that were pretreated with *N*-acetylcysteine (NAC), an antioxidant, displayed significantly reduced red and blue fluorescence, which confirmed the overproduction of O_2_^•–^ and ONOO^–^ in the lysosomes of HIRI hepatocytes. It is well known that O_2_^•–^ acts as the first ROS and triggers the production of other ROS, as such we anticipated that the total amount of ROS in the lysosomes of HIRI hepatocytes would be significantly increased and was not limited to O_2_^•–^ and ONOO^–^. We then extracted a large number of hepatocytes from the control and HIRI groups and separated the lysosomes from the two groups of cells using lysosome extraction kits. A 2.8-fold ROS level enhancement of the lysosomes in the HIRI group was detected using a ROS assay kit containing 2′,7′-dichlorodihydrofluorescein diacetate (DCFH-DA) a fluorescent probe for ROS (Figure 4E). These results confirm that lysosomal ROS content increases significantly during HIRI.

To investigate whether the local viscosity changes in lysosomes were regulated by lysosomal ROS fluctuations during HIRI, we simultaneously imaged ROS and lysosomal viscosity in the control and HIRI hepatocytes applying NP-V and DCFH-DA, which converted to fluorescent 2′,7′-dichlorofluorescein (DCF) at 525 nm under ROS oxidation.^57^ As shown in Figure 4B, both the red fluorescence of NP-V and the green fluorescence of DCF were increased in HIRI cells in sharp contrast to the control group, which again illustrated that lysosomal viscosity and intracellular ROS increased simultaneously in hepatocytes that suffered HIRI. Interestingly, reduced red fluorescence of NP-V and green fluorescence of DCF were observed in NAC-pretreated HIRI cells (Figure 4D and S13), which indicated that lysosomal viscosity was also decreased after intracellular ROS clearance. Therefore, the above results further elucidate that excess ROS in lysosomes can regulate the change in lysosomal viscosity during HIRI, which is a key factor responsible for lysosomal viscosity fluctuation.

### ROS Accumulation in Lysosomes Leads to Increased Lysosomal MDA Levels during HIRI

Inspired by the above results, we further explored how lysosomal ROS increased lysosomal viscosity levels during HIRI. It has been reported that ROS in lysosomes can lead to peroxidation of membrane polyunsaturated fatty acids and the formation of relatively stable MDA under oxidative stress.^13^ MDA, the final product of lipid peroxidation, plays a key role in causing lysosome degradation dysfunction by inducing protein cross-linking or reducing hydrolase activity.^13,58^ Therefore, we determined the MDA levels in lysosomes of the control and HIRI group hepatocytes by means of an MDA assay kit. As shown in Figure S14, MDA concentrations in the HIRI group lysosomes were about 4.0 times greater than that of the control group. The above data confirm that the accumulation of lysosomal ROS during HIRI causes the increase of MDA in lysosomes.

### Excessive MDA in Lysosomes Reduces Cathepsin B Activity during HIRI

Based on the above experiments, we found that ROS accumulation in lysosomes during HIRI resulted in excessive MDA formation. Next, we focused on the targets of excessive MDA on downstream proteins in the process of HIRI. As the most abundant lysosomal protease (up to 1 mM in lysosomes),^59^ cathepsin B controls protein degradation in lysosomes and maintains the microenvironmental homeostasis.^60^ Furthermore, the degradation activity of cathepsin B is dominated by cysteine and histidine residues at the functional active site, which are preferentially attacked by the highly active MDA.^58^ Therefore, cathepsin B was investigated as a potential downstream protein affected by MDA.

**Figure 5:**
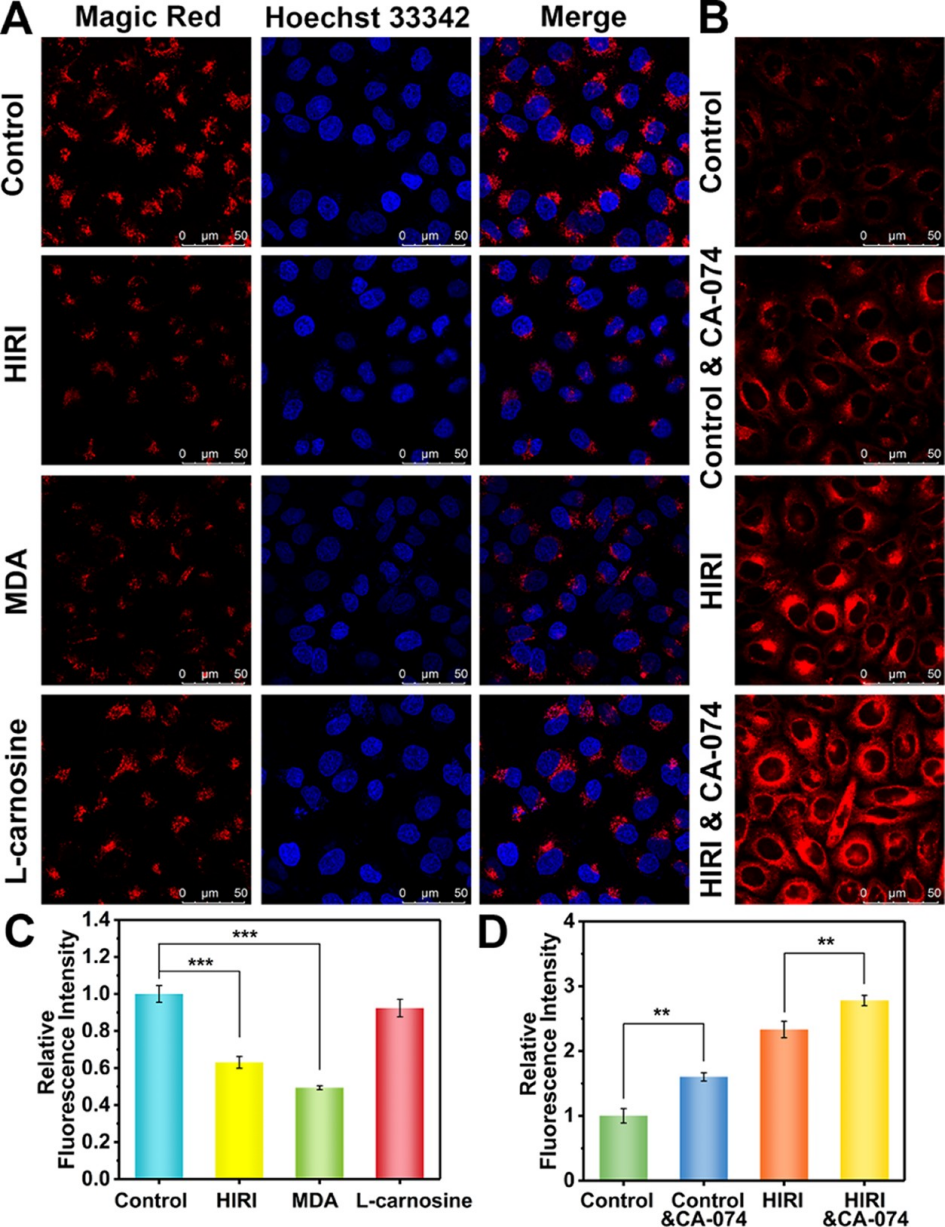
Cathepsin B activity assays and lysosomal viscosity detection of hepatocytes during different treatments. (A) Control hepatocytes, HIRI hepatocytes, 1 μM MDA-pretreated control hepatocytes, and 2 mM l-carnosine followed by 1 μM MDA-pretreated control hepatocytes were treated with Magic Red staining solution and Hoechst 33342 (1 μg/mL) for the analysis of cathepsin B activity. Red channel: Ex = 561 nm, collected from 610–713 nm. Blue channel: Ex = 405 nm, collected from 450–520 nm. (B) Control hepatocytes, 10 μM CA-074-pretreated control hepatocytes, HIRI hepatocytes, and 10 μM CA-074-pretreated HIRI hepatocytes were loaded with NP-V (20 μM) for fluorescence imaging of lysosomal viscosity. Ex = 633 nm collected from 640–820 nm. (C, D) Relative fluorescence intensity outputs of (A) and (B), respectively. The red fluorescence intensity of the control group was defined as 1. The data are expressed as the mean ± SD. **P < 0.01 and ***P < 0.001. Concordant results were obtained from three independent experiments.

Here, the activities of cathepsin B in hepatocytes subjected to various treatments were comprehensively evaluated using Magic Red cathepsin B assay kits.^15^ We found that the cathepsin B activity of the HIRI group was lower than that of the control group (Figure 5A,C), and after being stimulated by MDA, the activity of cathepsin B was significantly decreased in hepatocytes. After pretreatment with l-carnosine,^61^ an MDA scavenger, the activity of cathepsin B returned to a normal level. These observations illustrate that excessive MDA in lysosomes reduces cathepsin B activity during HIRI.

### Protein Mass Spectrometry Identifies the Addition Product of MDA to Cathepsin B

**Figure 6:**
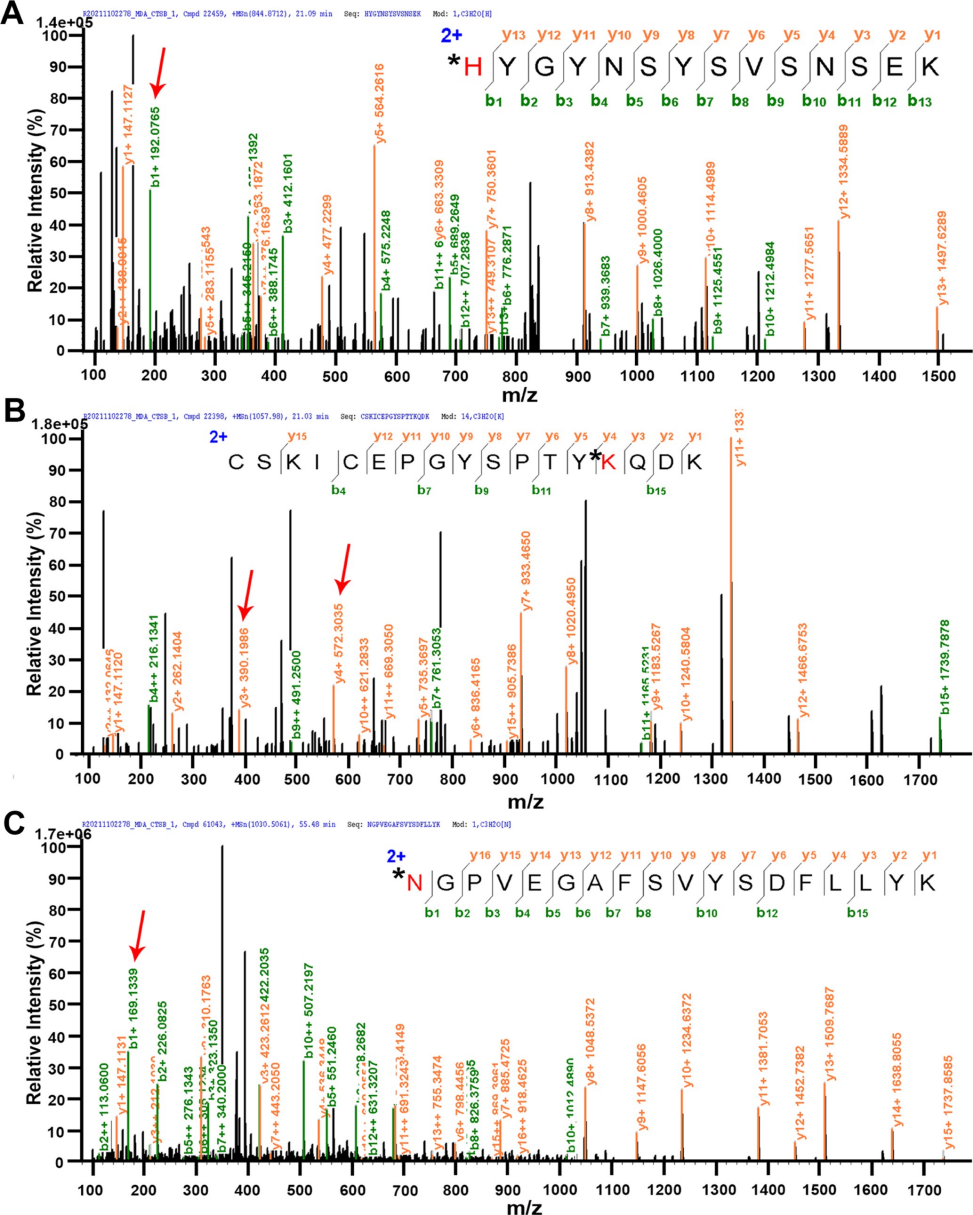
Proteomic analysis of the reaction of cathepsin B with MDA through LC-MS/MS. Representative MDA Schiff base adducts (+54 Da) on H224 (A), K220 (B), and N246 (C). Amino acids labeled with an * and red color denote modification by MDA. The relevant peaks due to amino acids modified by MDA (+54 Da) are highlighted by red arrows.

To explore the inactivation mechanism of cathepsin B caused by excessive MDA during HIRI, we performed proteomic analysis of cathepsin B using LC-MS to evaluate the post-translational modification of cathepsin B by MDA.^62^ As shown in Figures 6 and S15, we detected 11 types of Schiff base and dihydropyridine (DHP) MDA adducts at lysine, histidine, arginine, and asparagine sites. The predicted structures of the MDA-modified adducts of lysine, histidine, arginine, and asparagine in cathepsin B are summarized in Table S2.^62^ Together, these observations indicate that MDA reacts with lysine, histidine, arginine, and asparagine residues of cathepsin B and is probably responsible for the inactivation of cathepsin B.

### Cathepsin B Inactivation Results in Increased Lysosomal Viscosity during HIRI

Given that the reduced cathepsin B activity and increased lysosomal viscosity in HIRI were detected, we then evaluated the correlation between cathepsin B activity and lysosomal viscosity. Stronger red fluorescence was observed when normal cells were pretreated with a specific inhibitor of cathepsin B (CA-074)^60^ compared to the control group, indicating that lysosomal viscosity was indeed increased after cathepsin B inactivation (Figure 5B,D). CA-074-pretreated HIRI hepatocytes exhibited an intense red fluorescence signal, which confirmed that cathepsin B inactivation facilitated increased lysosomal viscosity in HIRI hepatocytes. Accordingly, this suggests that the inactivation of cathepsin B during HIRI is a major cause of the lysosomal viscosity increase.

### Potential Signaling Pathway

**Scheme 1:**
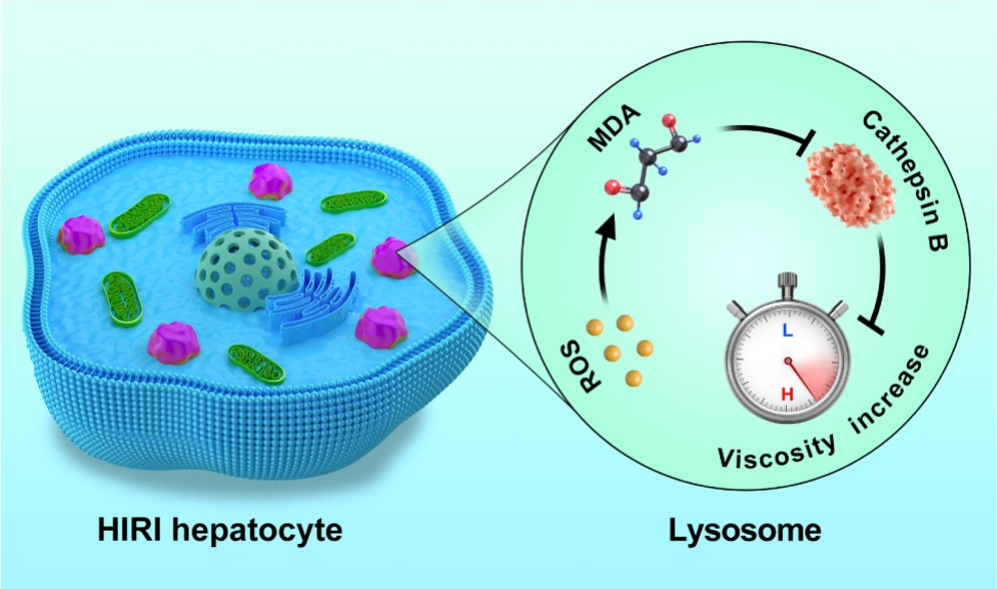
Schematic of Lysosomal ROS–MDA–Cathepsin B Cascade Signaling Pathway-Mediated Viscosity Change during HIRI

Based on the above experiments, we speculate that the lysosomal ROS–MDA–cathepsin B cascade signaling pathway mediates the viscosity changes during HIRI (Scheme 1). Taken together, when HIRI occurs, ROS accumulation in lysosomes produces excessive MDA through lipid peroxidation. Excessive MDA in lysosomes then covalently modifies cathepsin B and results in its inactivation, ultimately resulting in an increase in lysosomal viscosity and hepatocyte apoptosis and necrosis (Figure S16). The proposed signaling pathway fully elucidates the functionality of lysosomal viscosity as a reliable marker for HIRI, paving the way for accurate localization of HIRI.

### Precision Navigation of HIRI Liver Lesions Guided by NIR-II Fluorescence Imaging

**Figure 7:**
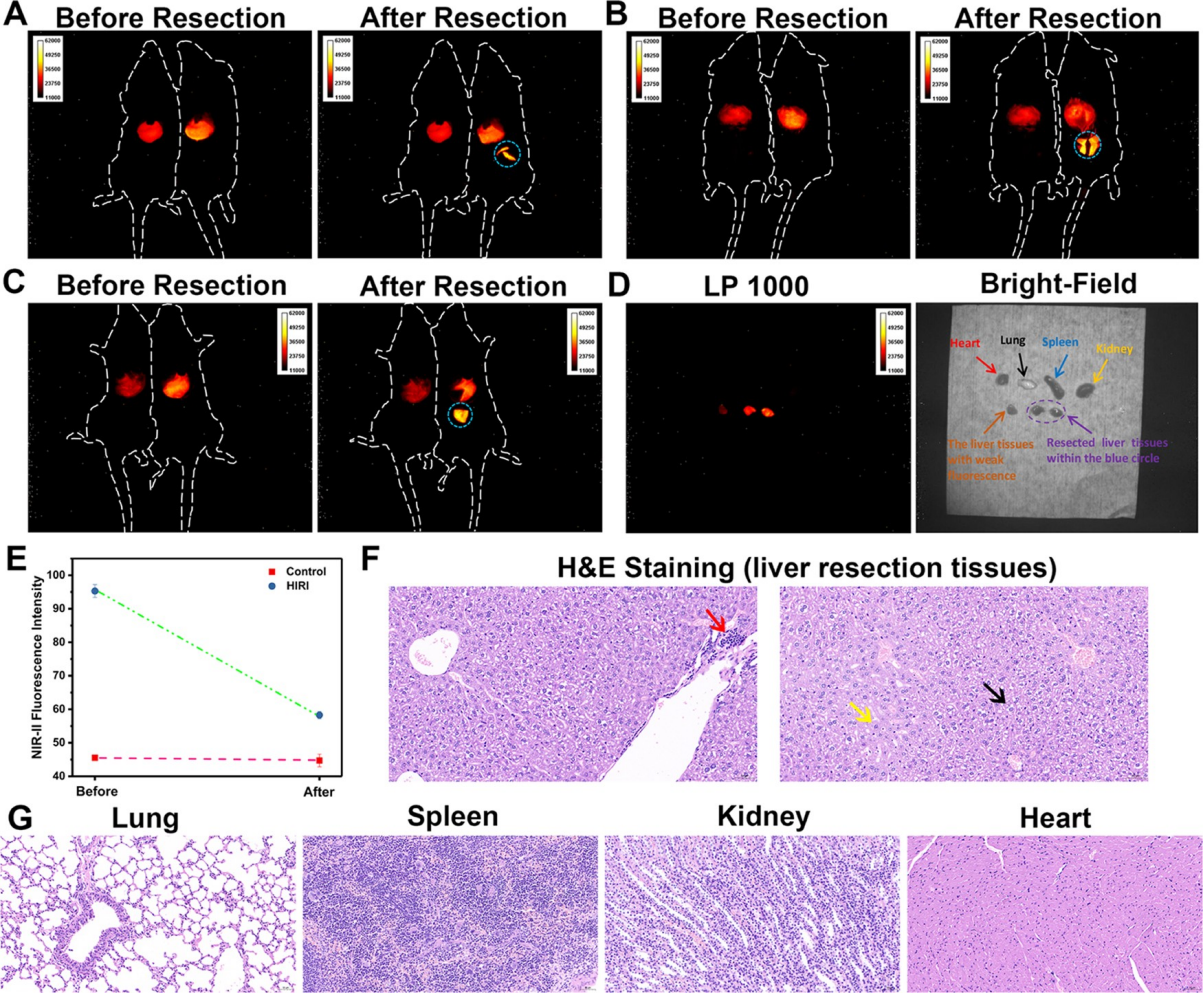
NIR-II fluorescence imaging in mice and NIR-II fluorescence image-guided HIRI lesions resection by NP-V. (A–C) Representative NIR-II fluorescence images for precise navigation and surgical resection of liver lesions in HIRI mice. Control mice are on the left and HIRI mice are on the right. The resected liver tissues in surgery with a high fluorescence intensity are in blue circles. The images were obtained under an excitation wavelength of 808 nm and a long-pass filter of 880 nm. (D) NIR-II fluorescence and bright-field images of the main organs (heart, spleen, lungs, kidneys, and resected liver tissues within the blue circle during the surgery (depicted by a purple arrow and a purple circle) and the liver tissues with weak fluorescence of HIRI mice) *in vitro* with a 1000 nm long-pass filter. (E) Relative fluorescence intensity output of (A)–(C). NIR-II fluorescence intensity output of the liver area in the control and HIRI mice before and after resection surgery. (F) H&E staining of liver resection tissues (within the blue circle) in HIRI mice. (G) H&E staining of lungs, spleen, kidneys, and heart tissues in HIRI mice. The data are expressed as the mean ± SD. Four mice in each group.

To further validate the effectiveness of lysosomal viscosity as a useful diagnostic reference marker for HIRI, NP-V was used to image and navigate HIRI lesions *in vivo* based on viscosity changes. Mice that were injected intravenously with NP-V were randomly divided into normal and HIRI groups. HIRI models were successfully established for mice, in which the portal vein and hepatic artery were clamped with hemostatic clips for 1 h ischemia, followed by 1 h reperfusion after releasing the hemostatic clips.^53^ Both groups were then transferred into a NIR-II fluorescence *in vivo* imaging system for imaging. As shown in Figure 7A–C, NIR-II images indicated that NP-V accumulated in the liver region of mice and exhibited good imaging contrast. Quantitative analysis indicated that the NIR-II fluorescence of the liver in the HIRI group of mice was 2.1 times higher than that of the control group of mice (Figure 7E). The SBR of NP-V for NIR-II imaging, defined as signals from the liver site relative to skin, was calculated as 2.0 for the control group. As for the HIRI group, the SBR was calculated as 3.7 (Figure S17). Based on the imaging analysis of the viscosity, we could distinguish the HIRI mice from normal mice according to the large fluorescence differences.

We found that the liver of the HIRI group of mice exhibited NIR-II fluorescence with a nonuniform intensity. We speculated that the parts with a high fluorescence intensity in the liver of the HIRI group were the liver lesion sites. To verify our hypothesis, a surgical operation on HIRI mice was performed. Benefiting from a high NIR-II fluorescence imaging resolution of 640 × 512 pixels, we used a scalpel to excise the part with a strong fluorescence intensity in the liver of HIRI mice. After excision, *in vivo* NIR-II fluorescence imaging was conducted again. From the fluorescence images after resection, we could distinctly see that the resected tissues within the blue circle exhibited strong NIR-II fluorescence compared with the unresected parts (Figure 7A–C). Quantitative analysis indicated that the NIR-II fluorescence signal in the liver of HIRI mice decreased significantly after resection of the strong fluorescence region (Figure 7E).

To evaluate the resected lesion tissues within the blue circle that suffered HIRI, we also resected some control group liver tissues and liver tissues with a weak fluorescence intensity of HIRI mice, plus the liver tissues within the blue circle of the HIRI group, which were excised during the surgery. Two comprehensive pathological examinations, including hematoxylin and eosin (H&E) staining and Masson staining, were performed on the three resected sites. H&E staining results confirmed that there were no pathological changes and no obvious inflammatory changes in normal liver tissues and the part of the liver tissues with a weak fluorescence intensity in the HIRI group (Figure S18). However, the resection tissues within the blue circle exhibited a large amount of ballooning degeneration of hepatocytes, swelling of cells, centralization of nuclei, vacuolation of the cytoplasm (black arrow), granular degeneration of hepatocytes around multiple blood vessels, loose and lightly stained cytoplasm with a fine granular shape (yellow arrow), and lymphocyte dotted infiltration (red arrow) with marked inflammatory changes (Figure 7F). Masson staining indicated that no fibrosis occurred in the three resected liver tissues (Figure S19). Histopathological examination results further confirmed that the resected tissues with a strong NIR-II fluorescence intensity indeed had obvious inflammatory infiltration, implying the location of HIRI lesions. As such, these results demonstrate that NP-V can provide effective information for intraoperative resection of lesion sites under the guidance of NIR-II fluorescence imaging.

Finally, HIRI mice were sacrificed, and organ imaging *in vitro* for the heart, spleen, lungs, kidneys, resected liver tissues within the blue circle, and liver tissues with weak fluorescence was performed to study the distribution of NP-V. *In vitro* imaging with a 1000 nm long-pass filter indicated that the heart, spleen, lung, and kidney tissues produced weak fluorescence, which was mainly concentrated in the liver region, and the fluorescence of the resection site in the purple oval was significantly higher than that of the liver region with weak fluorescence (Figure 7D). From H&E staining images, there were no pathological changes in the heart, spleen, lungs, and kidneys, which indicated that NP-V had good biocompatibility (Figure 7G). This application highlights the promising clinical potential of NP-V, which enables accurate navigation of HIRI liver lesions and effective differentiation between HIRI tissues and normal tissues. To confirm the conclusion that lysosomal viscosity is a marker of HIRI, an independent experiment was conducted. We extracted the lysosomes of the livers from the control and HIRI group mice using lysosome extraction kits. The lysosomal viscosities of the livers from the control and HIRI group mice were determined using an NDJ-8S rotational viscometer. The average lysosomal viscosity of the control group was 33.4 cp, and the average lysosomal viscosity of the HIRI group was 52.4 cp (Figure S20), providing evidence that lysosomal viscosity acts as a powerful marker of HIRI.

Lysosomal viscosity as a reliable reporter has several advantages for HIRI detection. Viscosity is such an important physical parameter of the microenvironment and leads to a wide range of signal coverage and obvious signal intensity changes. Molecular rotors based on fluorescence response to viscosity changes are faster and more sensitive than other “on–off” probes that depend on chemical reactions.^63^ In addition, we analyzed the causes of lysosomal viscosity and determined that it was a reliable biomarker of HIRI. First, lysosomal dysfunction and mitochondrial ROS-mediated autophagy flux damage are key events determining hepatotoxicity. Excessive ROS production leads to lysosome dysfunction. As lysosomes cannot effectively degrade damaged mitochondria through autophagy, the ROS production burden is exacerbated, leading to hepatotoxicity.^15^ Second, liver shear viscoelastic parameters increase significantly in HIRI.^17^ Taken together, these previous research suppositions have established that lysosomal viscosity is a powerful diagnostic indicator of HIRI for targeting and identifying the site of liver injury.

## Conclusions

In summary, we have developed a viscosity-activated NIR-II fluorescent probe NP-V for the precise highlighting of HIRI injury sites that enables the excising of HIRI inflammatory lesions under intraoperative fluorescence navigation in living mice. NP-V exhibited several advantages, including a 13-fold fluorescence response toward viscosity, a robust NIR-II emission, specific lysosomal-targeting ability, and good biocompatibility. Combining probes NP-V and LW-OTf, we revealed a lysosomal ROS–MDA–cathepsin B cascade signaling pathway-mediated viscosity variation during HIRI and determined that lysosomal viscosity was an ideal biomarker for HIRI navigation. More importantly, high SBR and superior brightness endowed NP-V with desirable performance for the precision navigation and resection of HIRI liver lesions in living mice, which was confirmed by histopathological examination. We envision that our proposed strategy based on lysosomal viscosity has the potential for further application in hepatology research to reveal the occurrence and progression of HIRI. We also anticipate that NP-V will be exploited to evaluate the therapeutic efficacy of HIRI drugs *in vivo*.

## Supporting Information Available

Additional experimental data including experimental details; synthesis of NP-V; photophysical properties; and fluorescence imaging of live cells (PDF)
