# Supplementary material for: Precision Navigation of Hepatic Ischemia–Reperfusion Injury Guided by Lysosomal Viscosity-Activatable NIR-II Fluorescence
Source: J Am Chem Soc. 2022 Jul 6;144(30):13586–99. doi: 10.1021/jacs.2c03832 (PMC9354259; doi:10.1021/jacs.2c03832)
Supplement: Supplementary file 1 — ja2c03832_si_001.pdf [file ja2c03832_si_001.pdf]

Supporting Information

Precision Navigation of Hepatic Ischemia−Reperfusion Injury Guided by Lysosomal Viscosity-Activatable NIR-II Fluorescence

Jihong Liu,[a] Wen Zhang,[a]* Chunmiao Zhou,[a] Mengmei Li,[a] Xin Wang,[a] Wei Zhang,[a] Zhenzhen Liu,[a] Luling Wu,[ab]* Tony D. James,[abc]* Ping Li,[a]* and Bo Tang[a]*

a. College of Chemistry, Chemical Engineering and Materials Science, Key Laboratory of Molecular and Nano Probes, Ministry of Education, Collaborative Innovation Center of Functionalized Probes for Chemical Imaging in Universities of Shandong, Institutes of Biomedical Sciences, Shandong Normal University, Jinan 250014, People's Republic of China.

b. Department of Chemistry, University of Bath, Bath, BA2 7AY, UK.

c. School of Chemistry and Chemical Engineering, Henan Normal University, Xinxiang 453007, People's Republic of China.

Email: zhangwen@sdnu.edu.cn, wllcyl@126.com, t.d.james@bath.ac.uk, lip@sdnu.edu.cn, tangb@sdnu.edu.cn

Table of Contents

Experimental Procedures S4
Materials and instruments S4
Preparation of ROS/RNS and MDA S4
The Förster-Hoffmann equation S5
Determination of quantum yield of NP-V S5
Cells culture S5
Cytotoxicity assays S6
Proteomic analysis S6
Hepatic ischemia–reperfusion injury models in cells S6
Hepatic ischemia–reperfusion injury models in mice S6
Evaluation of photobleaching of NP-V S7
Subcellular localization experiment of NP-V S7
Fluorescence imaging of viscosity under dexamethasone stimulation S7
Fluorescence imaging of lysosomal viscosity during HIRI S7
Fluorescence imaging of $ONOO^-$, $O_2^{\bullet -}$ during HIRI S7
Fluorescence imaging of intracellular ROS and lysosomal viscosity during HIRI S8
Lysosome extraction from hepatocytes S8
BCA protein content assays S8
ROS content assays in lysosomes of hepatocytes S8
MDA content assays in lysosomes of hepatocytes S9
Cathepsin B activity assays S9
Fluorescence imaging of lysosomal viscosity under CA-074 treatment S9
Determination of lysosomal viscosity of the livers from the control and HIRI group mice ... S9
Fluorescence imaging experiments in mice S10
Hematoxylin and eosin (H&E) staining of main organs in mice S10
Masson staining of resected liver tissues S10
Flow cytometry S11
Measurement of viscosity S11
Statistical analysis S11
Data availability S11
Results and Discussion S12
Synthesis of NP-V S12
Table S1 .... S13
Figure S1. .... S14
Figure S2. .... S14
Figure S3. .... S15
Figure S4. .... S15
Figure S5. .... S16
Figure S6. .... S16
Figure S7. .... S17
Figure S8. .... S18
Figure S9. .... S19

Figure S10. .....S19
Figure S11. .....S19
Figure S12. .....S20
Figure S13. .....S21
Figure S14. .....S22
Figure S15. .....S25
Figure S16. .....S26
Figure S17. .....S27
Figure S18. .....S27
Figure S19. .....S28
Figure S20. .....S29
Figure S21. .....S29
Figure S22. .....S30
Figure S23. .....S31
References S31

Experimental Procedures

Materials and instruments

1,1,2-trimethyl-1H-benz[e]indole was purchased from Shanghai yuanye Bio-Technology Co., Ltd. 1,3-propanesulfonate was purchased from Shanghai Macklin Biochemical Co., Ltd. N-[(3-(Anilinomethylene)-2-chloro-1-cyclohexen-1-yl)methylene]aniline monohydrochloride was purchased from Shanghai yuanye Bio-Technology Co., Ltd. Sodium hydrosulfide (NaHS) solution was prepared by dissolved NaHS in deionized water. CA-074 and dexamethasone were obtained from MedChemExpress. Recombinant Human Cathepsin B was purchased from ImmunoClone. ER-Tracker Green, Golgi-Tracker Green, LysoTracker Green, MitoTracker Green and Hoechst 33342 were purchase from Beyotime. BODIPY 493/503 was obtained from Invitrogen. *N*-acetyl-L-cysteine were purchased from Shanghai Aladdin Biochemical Technology Co., Ltd. Magic Red Cathepsin Assays was obtained from ImmunoChemistry Technologies, LLC. Bicinchoninic acid (BCA) Protein Assay Kit was purchased from Boster Biological Technology Co., Ltd. Lipid Peroxidation MDA Assay Kit was obtained from Beyotime. Lysosome Extraction Kit (HR0245, Bjbalb, China) and Reactive Oxygen Species Assay Kit (S0033S, Beyotime) were used. L-carnosine was purchased from Macklin Reagent (Shanghai, China).

Viscosity value was recorded by an NDJ-8S rotational viscometer. Absorption spectra were recorded on a UV-Visible spectrophotometer (Evolution 220, Thermo Scientific). Fluorescence spectra were obtained with a FLS-1000-SS-STM spectrometer (Edinburgh Instruments Ltd., England). MTT assay was performed using a Triturus microplate reader. Confocal imaging was performed on Leica SP8 high-resolution fluorescence microscope. NIR-II fluorescence imaging in mice was performed on a NIR-II Imaging System-H Series II 900/1700 equipped with 880 nm and 1000 nm long-pass filter. Hematoxylin-eosin (H&E) staining images were obtained with optical microscope (NIKON, Eclipse Ci-L) and Panoramic slice scanner (3DHISTECH, PANNORAMIC DESK/MIDI/250/1000) with the supporting software (3DHISTECH, CaseViewer2.4). Masson staining images were collected through Panoramic slice scanner (3DHISTECH, PANNORAMIC DESK/MIDI/250/1000) with the supporting software (3DHISTECH, CaseViewer2.2). ^{1}H NMR spectra were obtained at 400 MHz using Bruker NMR spectrometers, and ^{13}C NMR spectra were recorded at 100 MHz. The mass spectra were obtained using the Bruker Maxis ultra-high-resolution-TOF MS system. Proteomic analysis was performed by Q Exactive (Thermo Fisher) through LC-MS/MS. Flow cytometry was measured on an ImageStreamX Mark II flow cytometer (Merck).

Preparation of ROS/RNS and MDA

$O_2^{\bullet-}$

$O_2^{\bullet-}$ was produced from KO_2 in dry DMSO by an ultrasonic method. The concentration of $O_2^{\bullet-}$ was determined from the absorption at 250 nm (ε = 2682 M^{-1} cm^{-1}).

$ONOO^-$

0.6 M $NaNO_2$, 0.6 M HCl and 0.7 M H_2O_2 were added simultaneously to a 3 M NaOH solution at 0 ºC. The

concentration of peroxynitrite was determined using extinction coefficient of 1670 M^{-1} cm^{-1} at 302 nm in 0.1 M NaOH (aq.).

H_2O_2

H_2O_2 solutions were accessed by dilution of 30% hydrogen peroxide aqueous solution, the concentration was determined from the absorption at 240 nm (ε = 43.6 M^{-1} cm^{-1}).

TBHP

TBHP solutions were accessed by dilution of 70 % *tert*-butyl hydroperoxide aqueous solution.

$^{\bullet}OH$

$^{\bullet}OH$ (hydroxyl radical) was generated by the Fenton reaction of $FeCl_2$ (1.0 mM) and H_2O_2 (200 μM) in deionised water.

NO

NO (Nitric oxide) was obtained from a stock solution prepared by sodium nitroprusside.

MDA

MDA solution was prepared from 1,l,3,3-tetraethoxypropane (TEP) as literature procedure[1] and the concentration of MDA was measured by UV-vis absorption spectrum before use.[2]

The Förster-Hoffmann equation

The Förster-Hoffmann equation was utilized to correlate the relationship between the fluorescence emission intensity of NP-V and the solvent viscosity.

$$\log F = k \log \eta + C$$

in which η is medium viscosity, F is fluorescence emission intensity, C is the concentration and temperature constant, and k is the dye dependent constant.

Determination of quantum yield of NP-V

The quantum yield of NP-V was calculated in a similar manner to that described in previous publications.[3-5] ICG in DMSO was utilized as a reference (Φ_f = 0.13). The absorbance values at 808 nm of NP-V and ICG solutions at five different concentrations were measured and the fluorescence emission spectra were collected. The fluorescence emission spectra were integrated and plotted against the absorbance at 808 nm. The calculation equation of quantum yield was estimated as:

$$\Phi_{f,\ sam} = \Phi_{f,\ ref} \times (n_{sam}^2/n_{ref}^2)\ (K_{sam}/K_{ref})$$

Where $\Phi_{f,\ sam}$ is the fluorescence quantum yield of the sample, $\Phi_{f,\ ref}$ is the fluorescence quantum yield of reference. K is the slope of the integrated fluorescence intensity vs. absorbance plot, n_{sam} and n_{ref} are the refractive index.

Cells culture

Human hepatocytes (HL-7702) were purchased from the Cell Bank of the Chinese Academy of Sciences

(Shanghai, China). Hepatocytes were cultured in high-glucose DMEM supplemented with 10 % fetal bovine serum, 1 % penicillin and 1 % streptomycin (w v^{-1}) at 37 °C in a 5 % CO_2/95 % air MCO-15AC incubator (SANYO, Tokyo, Japan).

Cytotoxicity assays

3-(4,5-dimethylthiazol-2-yl)-2,5-diphenyltetrazolium bromide (MTT) assays were carried out to evaluate the toxicity of NP-V. Hepatocytes (10^6 cells mL^{-1}) were seeded into 96-well microtiter plates with total volumes of 200 μL $well^{-1}$. After 12 h of incubation, various concentrations of NP-V (0 M, 1×10^{-3} M, 1×10^{-4} M, 1×10^{-5} M, 1×10^{-6} M, 1×10^{-7} M, 1×10^{-8} M and 1×10^{-9} M) were added, and the hepatocytes were cultured for another 24 h. Afterwards, 20 μL of MTT solution (5 mg mL^{-1}) was added to each well. After 4 h, the MTT solution was removed, and 150 μL of DMSO was added to each well. Finally, the absorbance at 490 nm was measured using a Triturus microplate reader.

Proteomic analysis

Cathepsin B (1.0 mg mL^{-1}) was incubated for 24 h at 37 °C in buffer (10 mM PBS, pH 7.4) supplemented with 1.0 mM MDA. The Cathepsin B was then subjected to lyophilization and trypsin digestion. The peptides were isolated from the hydrolysate by solid-phase extraction on a C-18 column. Proteomic analysis was then performed through LC-MS/MS. The LC-MS/MS data were submitted to Mass spectrometry matching software (MASCOT) for analysis, to obtain the identification information of polypeptide. Mass additions of 54 and 134 Da were searched to identify MDA Schiff base adducts and dihydropyridine (DHP) type MDA adducts, respectively.[6]

Hepatic ischemia−reperfusion injury models in cells

Hepatic ischemia−reperfusion injury (HIRI) cell models were established by oxygen-glucose-serum deprivation/reperfusion. HL-7702 cells were cultured in DMEM (without glucose and serum) and deoxygenated sodium dithionite (0.5 mM) for 30 min. Subsequently, these cells were incubated with high glucose and serum DMEM (standard DMEM) in a 5 % CO_2 and 95 % O_2 atmosphere for 20 min.

Hepatic ischemia−reperfusion injury models in mice

Ten-week-old Kunming mice (females) were used. A HIRI mouse model was established by simulating liver surgery. The mice in HIRI group were given a laparotomy to expose the liver, and hepatic ischemia was induced by clamping the portal vein and hepatic artery of the median and the left lateral lobes of the liver with a microvessel clip, which induced partial (70 %) liver ischemia. One hour later, the vascular clamp was opened for one hour reperfusion. For normal group, the liver was exposed as a control. During the laparotomy surgery, the mice were anesthetized by inhaling isoflurane. All animal experiment methods were approved by Animal experiment ethical review committee of Shandong Normal University (Application number: AEECSDNU2021078).

Evaluation of photobleaching of NP-V

The photobleaching of NP-V in water was examined under continuous irradiation with an 808 nm laser (900 mW/cm^2) for 760 s. Upon irradiation of NP-V and ICG with an 808 nm laser (900 mW/cm^2), the absorption of NP-V and ICG at the absorption maximum wavelength were recorded in 40 s intervals. To evaluate the intracellular photostability of NP-V, HL-7702 cells stained with NP-V (20 μM) or ICG (20 μM) were excited with a 633 nm laser (intensity 32.5 %) of the confocal high-resolution fluorescence microscope for 460 s. Fluorescence images were captured under irradiation with the 633 nm laser after every 20 s irradiation.

Subcellular localization experiment of NP-V

HL-7702 cells were coincubated with NP-V (20 μM) and each corresponding commercial organelle-specific dye for 40 min. The cell culture medium of each group was removed, and all cells were washed with 1.0 mL of PBS three times before fluorescence imaging. Colocalization photographs were taken using a Leica SP8 high-resolution fluorescence microscope and Leica Application Suite X software.

Fluorescence imaging of viscosity under dexamethasone stimulation

HL-7702 cells were divided into four groups and pretreated with dexamethasone (5 μM) for 0, 5, 10 and 20 min, respectively. Subsequently, all the group cells were stained with NP-V (20 μM) for 25 min and then washed with 1.0 mL of PBS three times. Finally, the cells were subjected to confocal fluorescence imaging using a Leica SP8 high-resolution fluorescence microscope and Leica Application Suite X software.

Fluorescence imaging of lysosomal viscosity during HIRI

HL-7702 cells were divided into two groups (control and HIRI group). NP-V (20 μM) was added to the culture plates of two groups which were filled with 1 mL fresh complete medium for 25 min. After the incubation of NP-V for 25 min, the cell culture medium of all groups was removed, and the time point was defined as 0 min. For HIRI group, hepatocytes were cultured in DMEM (without glucose and serum) and deoxygenated sodium dithionite (0.5 mM) at 0−30 min. Afterwards, HIRI cells were incubated with high glucose and serum DMEM in a 5 % CO_2 and 95 % O_2 atmosphere at 31−50 min. Control group hepatocytes were cultured with high glucose and serum DMEM in a 5 % CO_2 and 95 % O_2 atmosphere at 0−50 min. Confocal fluorescence images were captured from the two groups of cells at time points of 10, 20, 30, 40 and 50 min.

Fluorescence imaging of $ONOO^-$, $O_2^{\bullet-}$ during HIRI

HL-7702 cells were divided into three groups. The control group cells were placed under normal culture conditions. The HIRI model group cells were treated with DMEM (no glucose and serum) and deoxygenated sodium dithionite (0.5 mM) for 30 min. Then, HIRI cells were incubated with standard DMEM in a 5 % CO_2 and 95 % O_2 atmosphere for 20 min. To investigate injury remediation, HL-7702 cells were pretreated with NAC (1 mM) for 1 h and then cultured with DMEM (without glucose and serum) and deoxygenated sodium dithionite (0.5 mM) for 30 min, followed by incubation with standard DMEM in a 5 % CO_2 and 95 % O_2

atmosphere for 20 min. All the groups were treated with 2.4 μM LW-OTf for 15 min before confocal imaging.

Fluorescence imaging of intracellular ROS and lysosomal viscosity during HIRI

HL-7702 cells were divided into three groups. The control group cells were placed under normal culture conditions. The HIRI model group cells were treated with DMEM (no glucose and serum) and deoxygenated sodium dithionite (0.5 mM) for 30 min. Then, HIRI cells were incubated with standard DMEM in a 5 % CO_2 and 95 % O_2 atmosphere for 20 min. For HIRI + NAC group, HL-7702 cells were pretreated with NAC (1 mM) for 1 h and then cultured with DMEM (without glucose and serum) and deoxygenated sodium dithionite (0.5 mM) for 30 min, followed by incubation with standard DMEM in a 5 % CO_2 and 95 % O_2 atmosphere for 20 min. All the group cells were co-stained with NP-V (20 μM) and DCFH-DA (10 μM) for 25 min before confocal imaging.

Lysosome extraction from hepatocytes

The lysosome extraction was performed using a Lysosome Extraction Kit (HR0245, Bjbalb, China). Hepatocytes were plated on culture flask, cultured with high-glucose DMEM (2 mL) for 36–72 h prior to the day of the experiment and maintained at 37 °C in a 5% CO_2 atmosphere until they reached 80–90% confluence. Then the hepatocytes were divided into two groups. The control group was cultured under normal culture condition. The HIRI group was subjected to ischemia for 30 min and reperfusion for 20 min. All treated cells were trypsinized and centrifuged for 5 min at 500 × g. The cells were resuspended in PBS buffer and centrifuged for 5 min at 400 × g. 400 μL of lysosome extraction solution A was added to the cells and the cells were put on the ice for 10 min. Dounce homogenizer was used to homogenate 30 times. The homogenate was centrifuged for 5 min at 1000 × g and liquid supernatant was collected. Then the liquid supernatant was centrifuged for 10 min at 3000 × g and liquid supernatant was collected. The liquid supernatant was centrifuged for 10 min at 5000 × g and liquid supernatant was collected. The liquid supernatant was centrifuged for 20 min at 30000 × g and sediment was collected. 400 μL of lysosome extraction solution B was added to the sediment and mixed. The mixed solution was centrifuged for 20 min at 30000 × g and sediment was collected. The resulted sediment was resuspended in lysosome preserving solution as lysosome sample.

BCA protein content assays

The BCA protein content assays were carried out by BCA Protein Assay Kit (Boster). The above lysosome samples were lysed in the lysis buffer. Then 25 μL of reference sample and the sample to be tested were added in microplate, respectively. 200 μL of BCA working solution was added in each well and vibrate to mix sufficiently. The microplate was covered and incubated for 30 min at 37 °C. After cooling to room temperature, The absorbance at 562 nm was measured using a Triturus microplate reader. The protein concentrations of samples were determined according to the standard curve of reference sample and the dilution ratio of samples.

ROS content assays in lysosomes of hepatocytes

The ROS contents were detected using Reactive Oxygen Species Assay Kit (S0033S, Beyotime). The above

lysosomes were collected and resuspended in DCFH-DA. The lysosomes were incubated at 37 °C for 20 min and mixed at intervals of 3–5 min. The emission at 528 nm under 485 nm excitation was detected using a Triturus microplate reader.

MDA content assays in lysosomes of hepatocytes

The MDA contents in lysosome per unit protein weight were calculated combined with BCA protein content assay kit and MDA Assay Kit. The above lysosome samples were collected and lysed in the lysis buffer. After lysis, the samples were centrifuged at 10000×g for 10 min and the liquid supernatant was collected. The protein contents of samples were determined using a BCA Protein Assay Kit to calculate the MDA content in lysosome per unit protein weight. 0.37 % of thiobarbituric acid (TBA) stock solution was prepared. MDA detection working solution was made through addition of TBA stock solution and TBA diluent and antioxidant. Reference samples were diluted and the concentration of 1 μM, 2 μM, 5 μM, 8 μM and 10 μM were prepared. Reference samples and samples to be tested are added with 200 μL MDA detection working solution and mixed. After mixing, the solution was heated at 100 °C for 15 min and cooled to room temperature. The solution was centrifuged for 10 min at 1000×g. 200 μL of the solution was added in 96-well plates and the absorptance of 532 nm was detected.

Cathepsin B activity assays

Cathepsin B activity assays were performed using a Magic Red Cathepsin Assays kit (immunochemistry). HL-7702 cells were divided into four groups. The control group was incubated with standard DMEM as normal. HIRI model group was cultured in DMEM (glucose and serum free) with deoxygenated sodium dithionite (0.5 mM) for 30 min and then incubated with high glucose and serum DMEM in a 5 % CO_2 and 95 % O_2 atmosphere for 20 min. MDA group was pretreated with 1 μM MDA for 30 min. The L-carnosine group was pretreated 2 mM L-carnosine for 30 min followed by 1 μM MDA for 30 min. All the group cells were loaded with Magic Red staining solution for 30 min and Hoechst 33342 (1 μg/mL) for a subsequent 15 min.

Fluorescence imaging of lysosomal viscosity under CA-074 treatment

HL-7702 cells were divided into four groups. The control group cells were placed under normal culture conditions. Control & CA-074 group cells were pretreated with 10 μM CA-074 for 3 h. HIRI model group cells were cultured in DMEM (without glucose and serum) with deoxygenated sodium dithionite (0.5 mM) for 30 min and high glucose and serum DMEM in a 5 % CO_2 and 95 % O_2 atmosphere for 20 min. HIRI & CA-074 group was pretreated with 10 μM CA-074 for 3h and cultured in DMEM (without glucose and serum) with deoxygenated sodium dithionite (0.5 mM) for 30 min followed by high glucose and serum DMEM in a 5 % CO_2 and 95 % O_2 atmosphere for 20 min. Afterwards, all the cell groups were incubated with NP-V (20 μM) for 25 min.

Determination of lysosomal viscosity of the livers from the control and HIRI group mice

A HIRI mouse model was established following the above method. The lysosomes of the livers from the control

and HIRI group mice were extracted using Lysosome Extraction Kit (HR0245, Bjbalb, China). Subsequently, the separated lysosomes of the livers from both groups were lysed in the lysis buffer. After lysis, the lysosomes samples were centrifuged at 10000×g for 10 min and the liquid supernatant was collected. The viscosity of lysosome samples from both groups were determined using an NDJ-8S rotational viscometer.

Fluorescence imaging experiments in mice

NP-V with a dose of 0.3 mM was intravenously injected into the mice through the tail vein, after 15 min, mice were randomly divided into normal group and HIRI group. Then the mice in HIRI group underwent a laparotomy surgery, hepatic ischemia was induced by clamping the portal vein and hepatic artery of the median and the left lateral lobes of the liver with a microvessel clip, inducing partial (70 %) liver ischemia. One hour later, the vascular clamp was opened for one hour reperfusion. The mice in the normal group were subjected to a laparotomy and the livers were exposed. All mice were transferred in NIR-II Imaging System-H Series II 900/1700 equipped with 880 nm and 1000 nm long-pass filter for *in vivo* NIR-II imaging and image-guided HIRI lesions resection. The excised liver tissues were subjected to histopathological examination. The mice were anesthetized with isoflurane prior to injection and during imaging.

Hematoxylin and eosin (H&E) staining of main organs in mice

After NIR-II fluorescence image-guided HIRI lesions resection, the mice in the HIRI group were sacrificed and dissected to isolate the spleen, lungs, heart and kidneys. In addition, the liver excision tissues within the blue circle during the surgery and some HIRI group liver tissues with weak fluorescence as well as some control group liver tissues were also prepared. All isolated organs were fixed in 4 % paraformaldehyde for tissue staining. The samples were dehydrated, embedded, sectioned and stained by hematoxylin and eosin. The sections were imaged through optical microscope (NIKON, Eclipse Ci-L) and Panoramic slice scanner (3DHISTECH, PANNORAMIC DESK/MIDI/250/1000) with the supporting software (3DHISTECH, CaseViewer2.4).

Masson staining of resected liver tissues

After NIR-II fluorescence image-guided HIRI lesions resection, the liver excision tissues within the blue circle during the circle and some HIRI group liver tissues with weak fluorescence as well as partial liver tissues in the control group were fixed in 4 % paraformaldehyde for tissue staining. The samples were dehydrated, embedded, sectioned and stained by acid magenta-aniline blue. The images were collected through Panoramic slice scanner (3DHISTECH, PANNORAMIC DESK/MIDI/250/1000) with the supporting software (3DHISTECH, CaseViewer2.2). After imaging, Image-Pro Plus 6.0 analysis software was used to uniformly take pixel area as the standard unit. The collagen pixel area and corresponding tissue pixel area in each section were measured respectively, and the positive area ratio was calculated as collagen pixel area/tissue pixel area×100.

Flow cytometry

Hepatocytes were seeded on six-well plates, cultured with high-glucose DMEM (2 mL) for 36–72 h prior to the day of the experiment and maintained at 37 °C in a 5% CO_2 atmosphere until they reached 80–90% confluence. Prior to flow cytometry, the hepatocytes under various treatments in each well were washed with PBS and trypsinized with 500 μL of 0.05% trypsin for 1 min. The trypsin was discarded, and 1 mL of high-glucose DMEM was added to each well. The cells were then centrifuged at 1000 rpm for 5 min, and the medium was discarded. The cells were resuspended in 1.5 mL of PBS and again centrifuged at 1000 rpm for 5 min. Next, each pellet was incubated with 195 μL of Annexin V-FITC binding buffer, 5 μL of Annexin V-FITC and 10 μL of propidium iodide (PI) at room temperature in the dark for 20 min. After incubation, the hepatocytes were centrifuged at 1000 rpm for 5 min and resuspended in 200 μL of PBS. After these procedures, the hepatocytes were subjected to flow cytometry. The fluorescence signals in the FITC channel and PI channel were measured for 5000 cells on an ImageStreamX Mark II flow cytometer (Merck). The data were analyzed using IDEAS software version 6.2.

Measurement of viscosity

The solution of NP-V (final concentration 5 μM) with different viscosity were obtained by adding the stock solution (2 mM) of NP-V to the mixture (2 mL) of water-glycerol mixture with different volume proportions. The solutions were shaken constantly for 1 h, and after standing for 30 min to eliminate air bubbles, their fluorescence were measured. The viscosity of the water-glycerol mixture in different proportions at (20 ± 0.1) °C is listed in Table S1.

Statistical analysis

All data are expressed as the mean ± S.D. The data under each condition were accumulated from at least three independent experiments. For each experiment, unless otherwise noted, n represents the number of individual biological replicates. For each biological replicate and for all *in vitro* and *ex vivo* studies, $n \geq 3$. The Student's t test was used for comparisons between two groups of experiments. Statistically significant P values are indicated in Figures and/or legends as ***$P < 0.001$, **$P < 0.01$.

Data availability

All relevant data that support the findings of this study are available from the corresponding author upon reasonable request.

Results and Discussion

Synthesis of NP-V

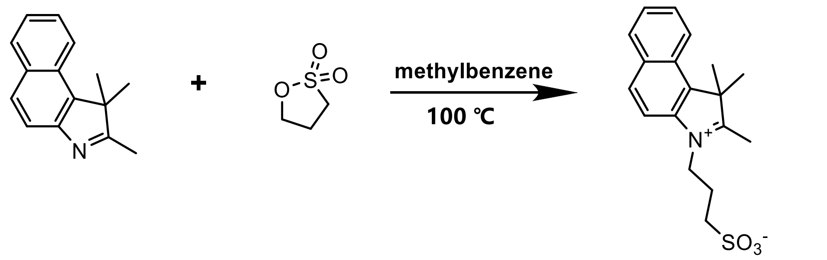

indolium salt

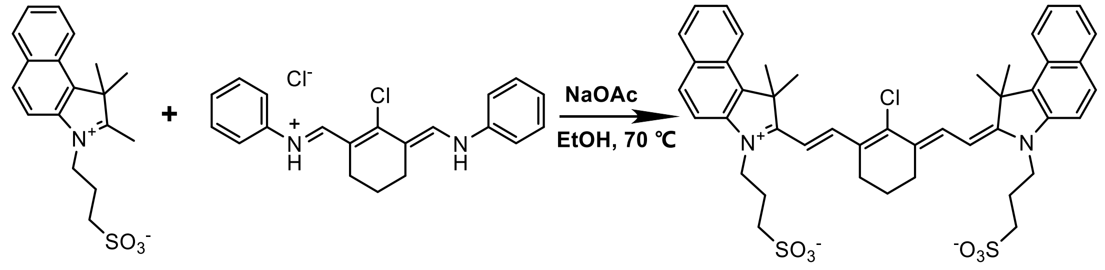

NP-V

Synthesis of indolium salt

Under nitrogen protection, 1,1,2-trimethyl-1H-benz[e]indole (1.046 g, 5.0 mM) was added to methylbenzene (10 mL), followed by the addition of 1,3-propanesulfonate (0.439 mL, 5.0 mM). The mixture was refluxed at 100 °C for 18 h, then allowed to cool down to room temperature. Diethyl ether was added to the resulting solution to precipitate a gray solid. The solid was collected by vacuum filtration and washed with acetone, to give the indolium salt as a gray solid (1.486 g, 4.5 mM, 90%). HRMS data, m/z calculated for $[C_{18}H_{20}NO_3S^-]$, 330.1169 found 330.1131.

Synthesis of NP-V

Indolium salt (0.990 g, 3 mM), Vilsmeier–Haack reagent (0.359 g, 1 mM) and anhydrous sodium acetate (0.328 g, 4 mM) were dissolved in absolute ethanol (20 mL) and the entire mixture was refluxed at 70 °C for 20 h under a nitrogen atmosphere. The ethanol was removed under reduced pressure washed with ether, and then purified by recrystallization from methanol and ether. The crude was subsequently purified by thin layer chromatography, eluting with dichloromethane/methanol (9:1, v/v) to afford NP-V as a green solid (0.247 g, 0.31 mM, 31%). HRMS data, m/z calculated for $[C_{44}H_{46}ClN_2O_6S_2^-]$, 797.2491 found 797.2484. ^{1}H NMR (400 MHz, DMSO-d_6): δ 8.38 (d, J = 12 Hz, 2H), 8.30 (d, J = 8 Hz, 2H), 8.10 (d, J = 12 Hz, 2H), 8.06 (d, J = 8 Hz, 2H), 7.87 (d, J = 8 Hz, 2H), 7.66 (t, J = 8 Hz, 2H), 7.52 (t, J = 8 Hz, 2H), 6.57 (d, J = 16 Hz, 2H), 4.52 (t, J = 8 Hz, 4H), 2.79 (t, J = 4 Hz, 4H), 2.65 (t, J = 4 Hz, 4H), 2.10 (m, 4H), 1.96 (s, 12H), 1.87 (m, 2H). ^{13}C NMR

(100 MHz, DMSO-d_6): δ 173.84, 147.81, 142.67, 140.27, 134.07, 131.93, 130.94, 130.41, 128.22, 127.94, 127.06, 125.41, 122.74, 112.30, 102.04, 51.14, 49.05, 48.24, 43.51, 27.51, 26.52, 24.23, 21.09.

Table S1. The viscosity of the water-glycerol mixture in different proportions at (20 ± 0.1) °C.

water (v%)	glycerol (v%)	viscosity (cP)	water (v%)	glycerol (v%)	viscosity (cP)
100	0	3.00	35	65	19.20
90	10	4.50	30	70	39.60
80	20	5.25	25	75	53.60
70	30	6.80	20	80	78.90
60	40	8.90	15	85	142.0
55	45	9.20	10	90	241.0
50	50	11.65	5	95	460.0
40	60	14.45			

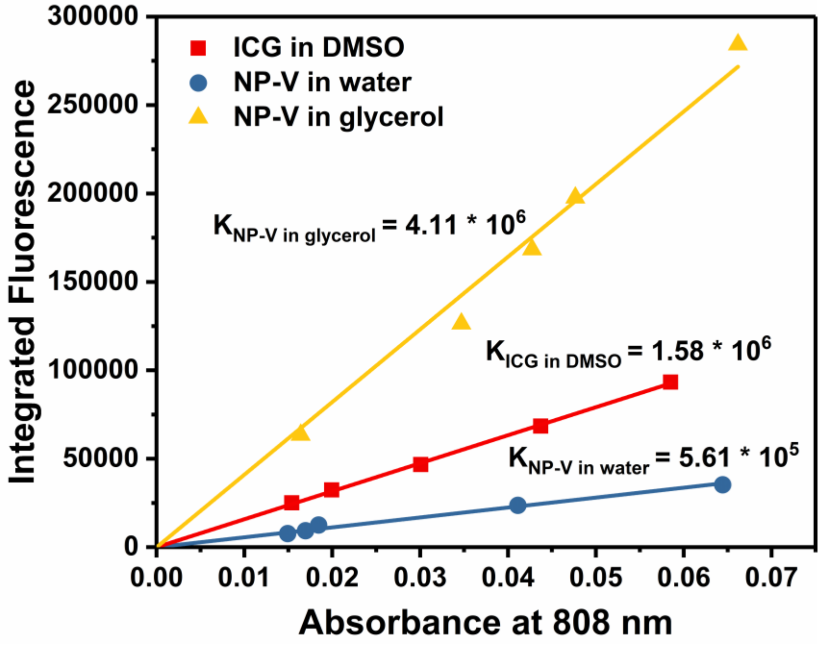

Figure S1. Calculated quantum yields of NP-V in water and glycerol. The plot of the fluorescence intensity of NP-V in water and glycerol at five different concentrations was used to calculate quantum yield by comparing the slopes of the linear fits. λ_{ex} = 808 nm, collected 810−1000 nm.

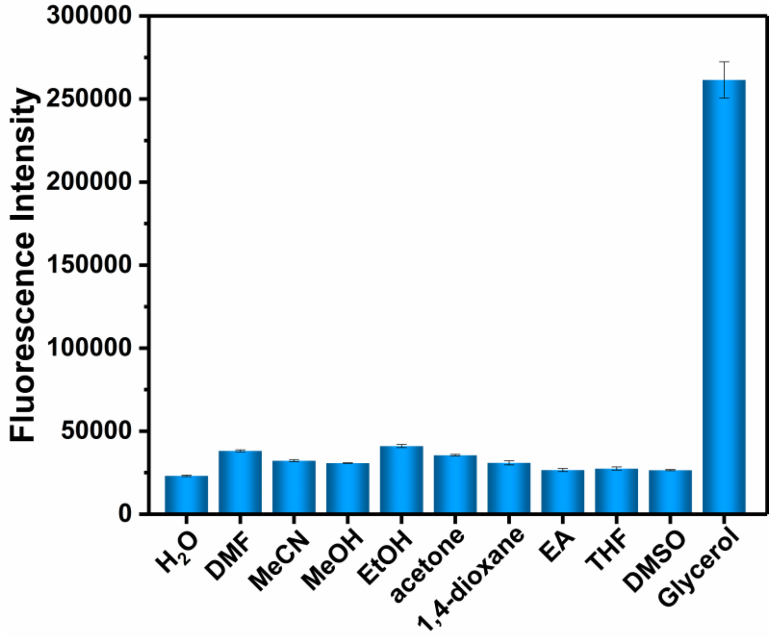

Figure S2. Fluorescence behavior of NP-V (5 μM) in different polar solvents (H_2O, DMF, MeCN, MeOH, EtOH, acetone, 1,4-dioxane, ethyl acetate, THF, DMSO, 95 % glycerol). $\lambda_{ex/em}$ = 808/864 nm.

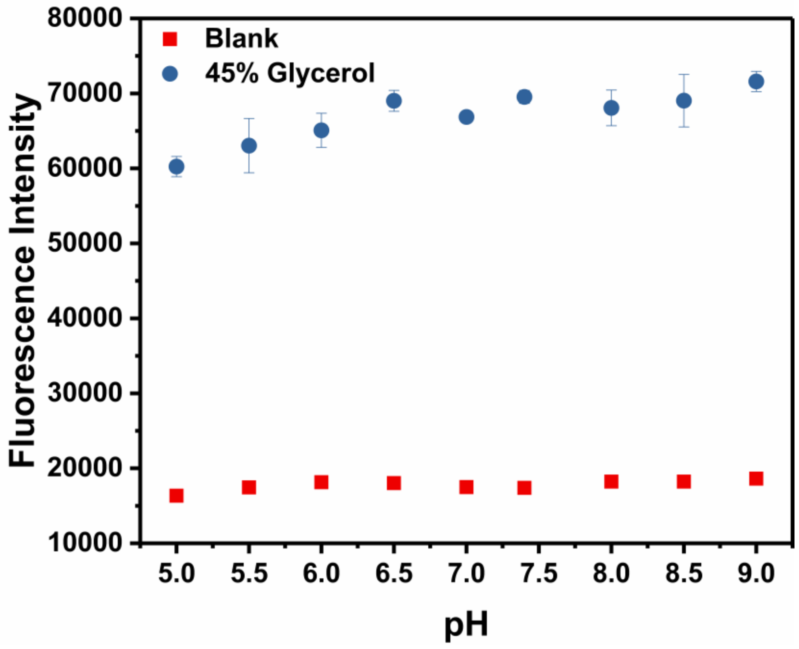

Figure S3. Fluorescence spectra of NP-V (5 μM) in PBS buffer (10 mM, red squares) and after the addition of glycerol (45%, blue circles) at various pH's. $\lambda_{ex/em}$ = 808/864 nm.

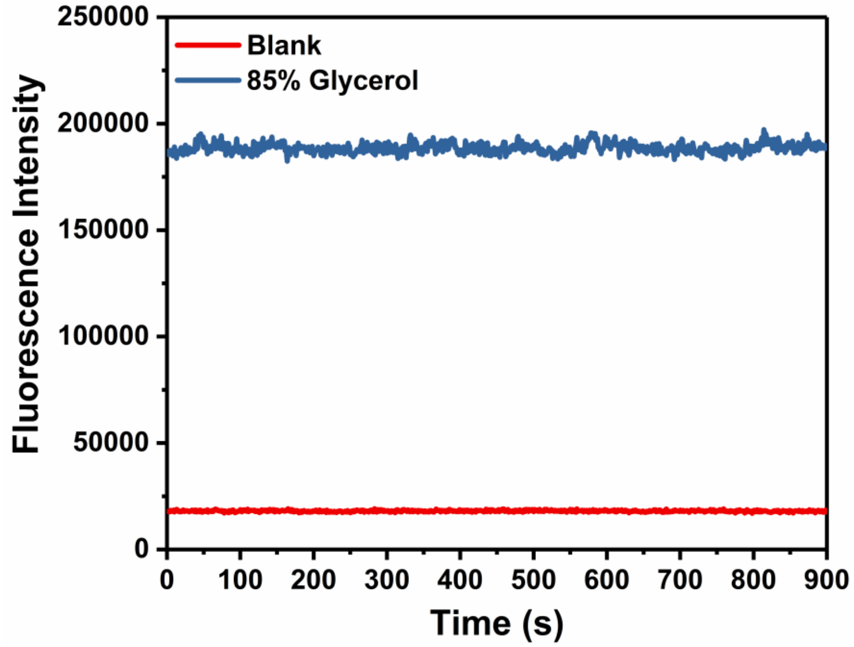

Figure S4. Time course for changes in the fluorescence of NP-V (5 μM) in PBS buffer (10 mM, red curve) and in glycerol (85%, blue curve). $\lambda_{ex/em}$ = 808/864 nm.

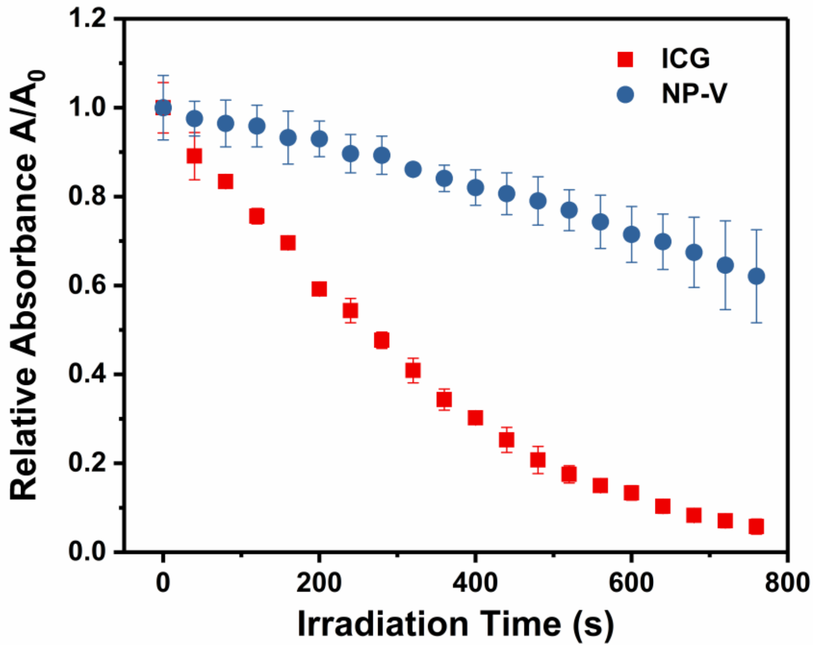

Figure S5. Photobleaching of NP-V and ICG in water. Relative absorbance of NP-V (5 μM) and ICG (5 μM) under irradiation with an 808 nm laser (900 mW/cm^2) after different irradiation times. Maximal absorbance was recorded and A_0 was defined as the absorbance at 0 s.

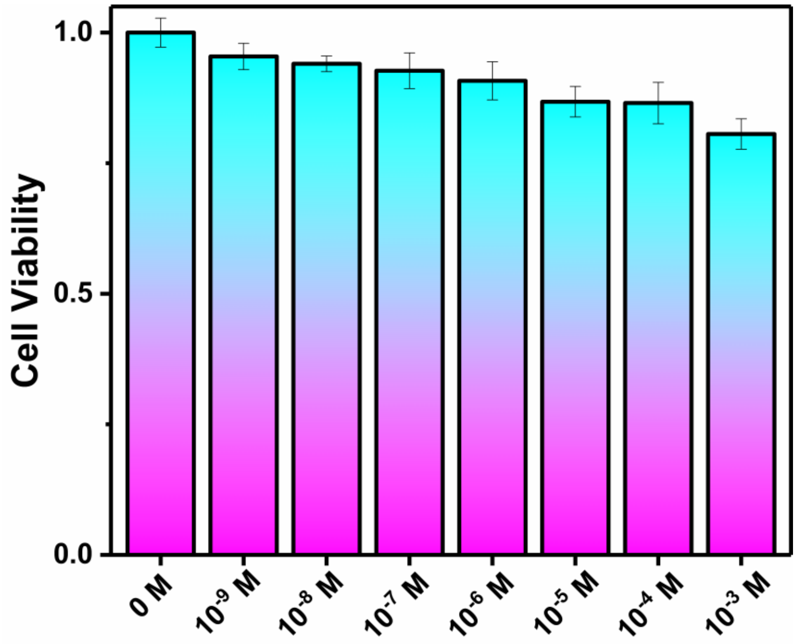

Figure S6. Cell toxicity of NP-V towards HL-7702 cells with an incubation time of 24 h. Error bar represents s.d.

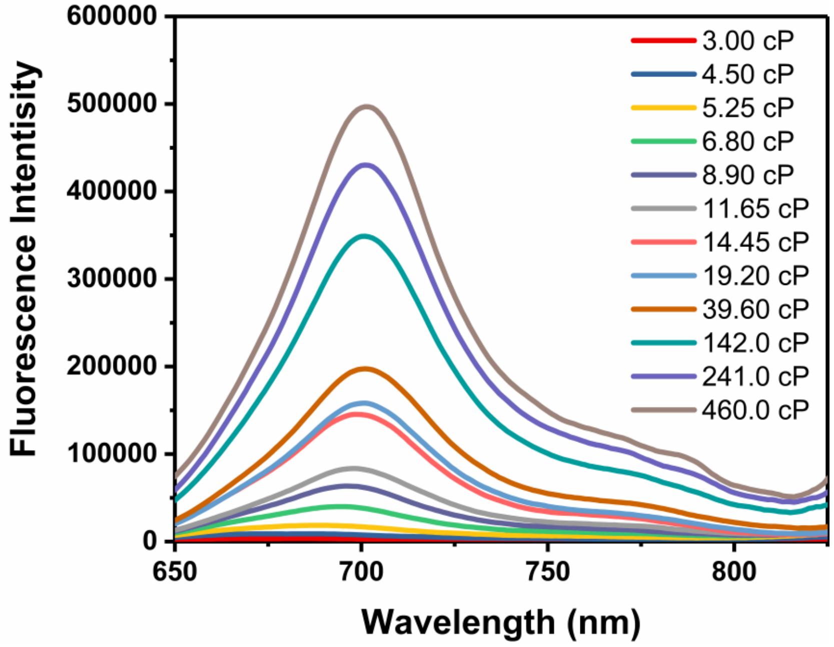

Figure S7. Fluorescence spectra of NP-V (5 μM) in water/glycerol mixtures with different viscosities (3.0−460.0 cP). λ_{ex} = 633 nm.

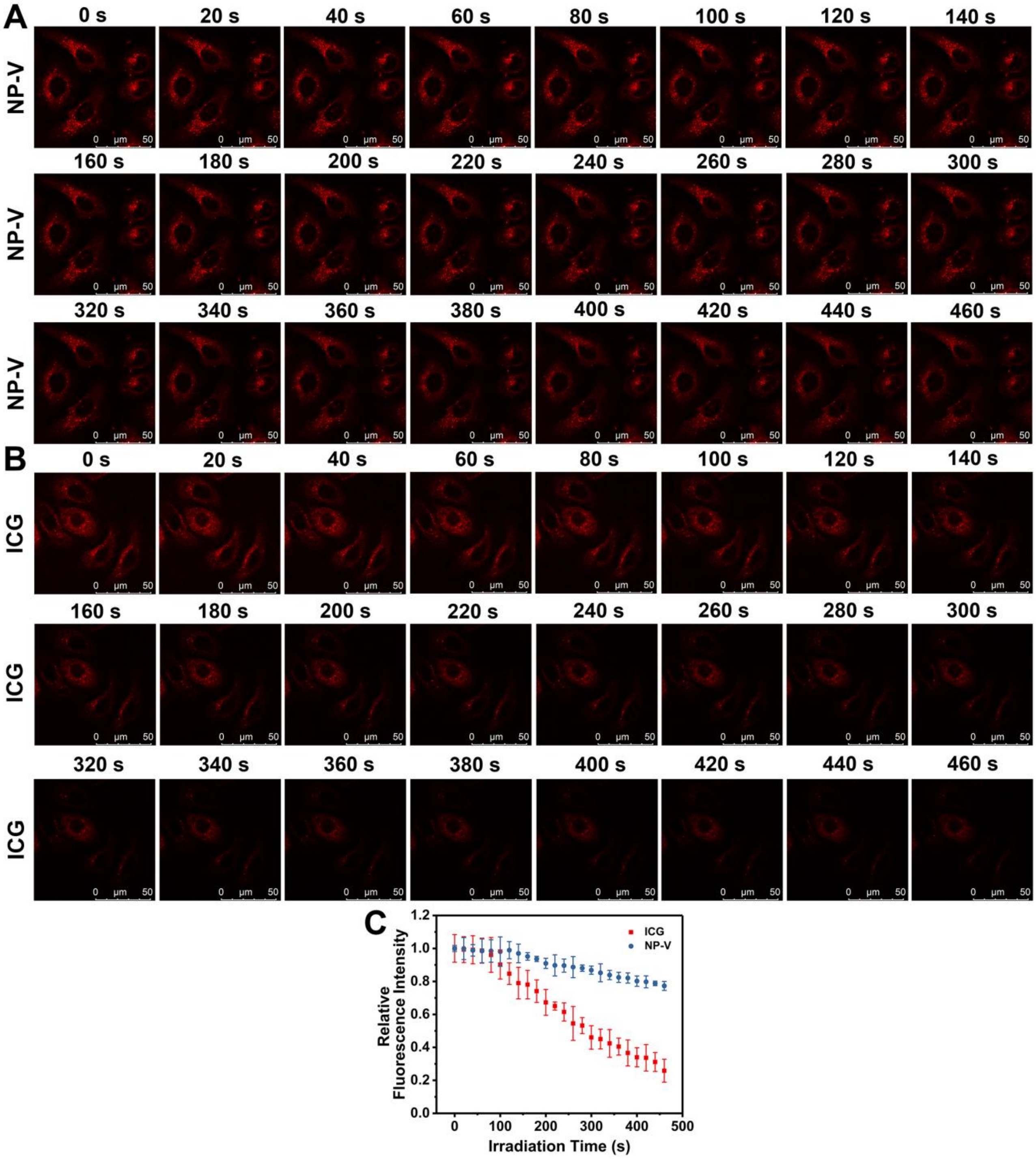

Figure S8. Intracellular photobleaching of NP-V and ICG under irradiation with a 633 nm laser (intensity 32.5 %) in HL-7702 cells. A. Fluorescence images of HL-7702 cells stained with 20 μM NP-V were captured after every 20 s irradiation. B. Fluorescence images of HL-7702 cells stained with 20 μM ICG were captured after every 20 s irradiation. C. The relative fluorescence intensity output of A and B. Normalization was carried out against the initial fluorescence intensity. Ex= 633 nm, collected 640−820 nm.

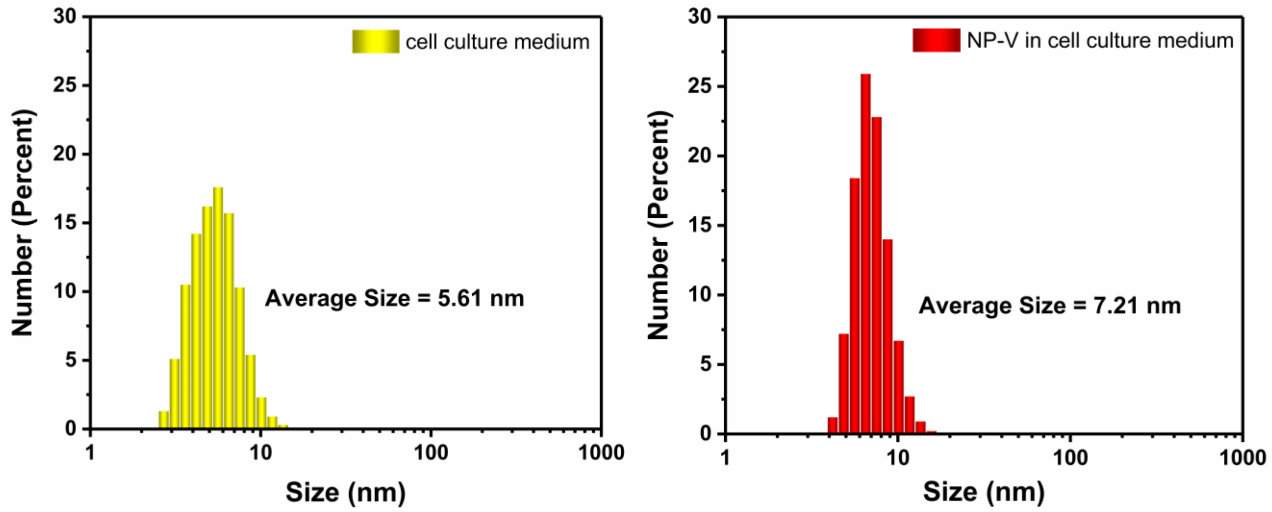

Figure S9. DLS size distribution of cell culture medium and NP-V (20 μM) in cell culture medium. Average size was calculated from three independent experiments.

Sample	Zeta potential (mV)
cell culture medium	-4.88
NP-V in cell culture medium	-6.43

Figure S10. Zeta potentials of cell culture medium and NP-V (20 μM) in cell culture medium.

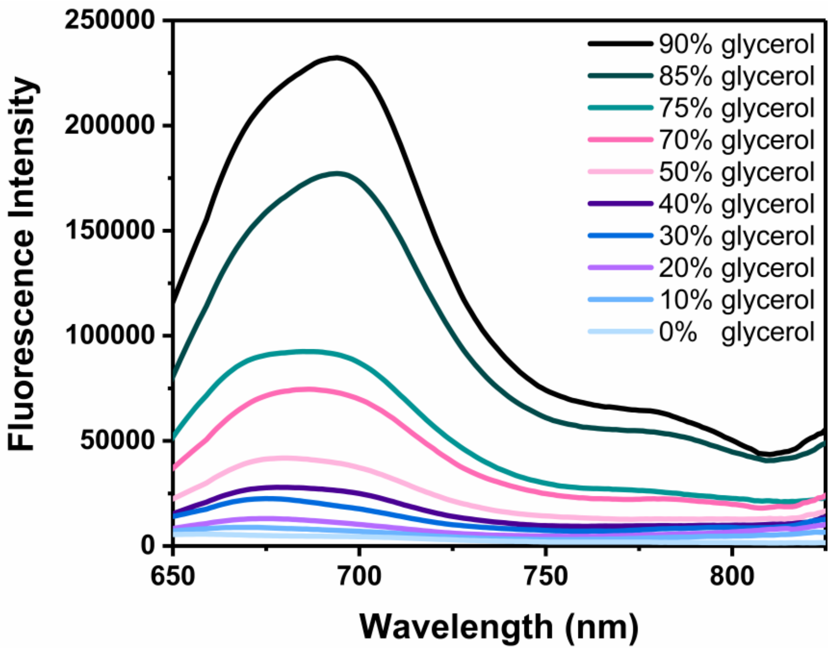

Figure S11. Fluorescence spectra of NP-V (5 μM) in cell culture medium/glycerol mixtures in different proportions. λ_{ex} = 633 nm.

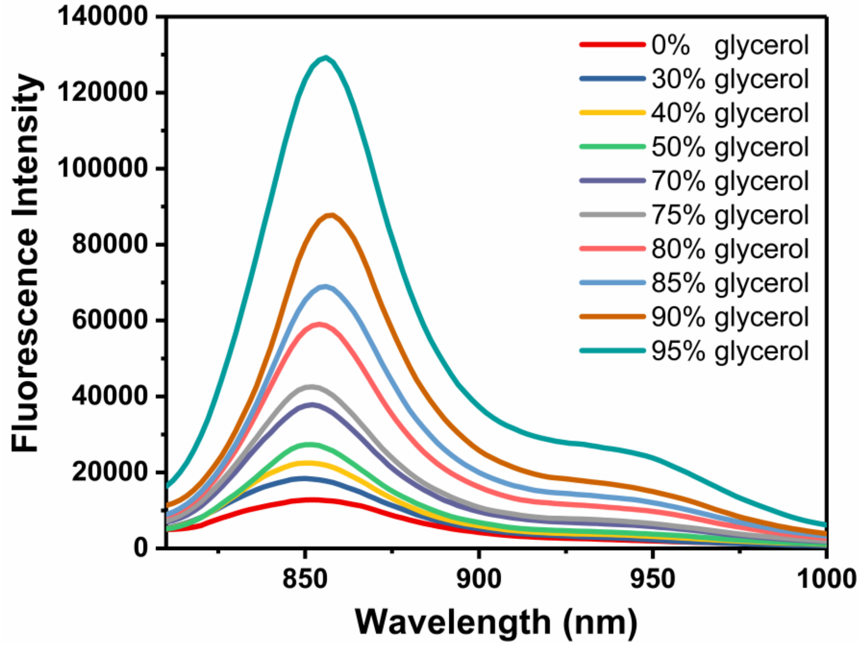

Figure S12. Fluorescence spectra of NP-V (5 μM) in cell culture medium/glycerol mixtures in different proportions. λ_{ex} = 808 nm.

Experimental procedure for Figure S13

HL-7702 cells were divided into three groups. Control group cells were placed under normal culture conditions. The HIRI model group cells were treated with DMEM (no glucose and serum) and deoxygenated sodium dithionite (0.5 mM) for 30 min. Then, HIRI cells were incubated with standard DMEM in a 5 % CO_2 and 95 % O_2 atmosphere for 20 min. The HIRI + NAC group cells were incubated with NAC (1 mM) for 1 h, followed by culturing with DMEM (without glucose and serum) and deoxygenated sodium dithionite (0.5 mM) for 30 min, and then cultured with standard DMEM in a 5 % CO_2 and 95 % O_2 atmosphere for 20 min. All group cells were stained with NP-V (20 μM) for 25 min before confocal imaging. The cell culture medium of each group was removed, and all cells were washed with 1.0 mL of PBS three times before fluorescence imaging. One-photon confocal photographs were taken imaged by Leica SP8 high-resolution fluorescence microscope equipped with the Leica Application Suite X software.

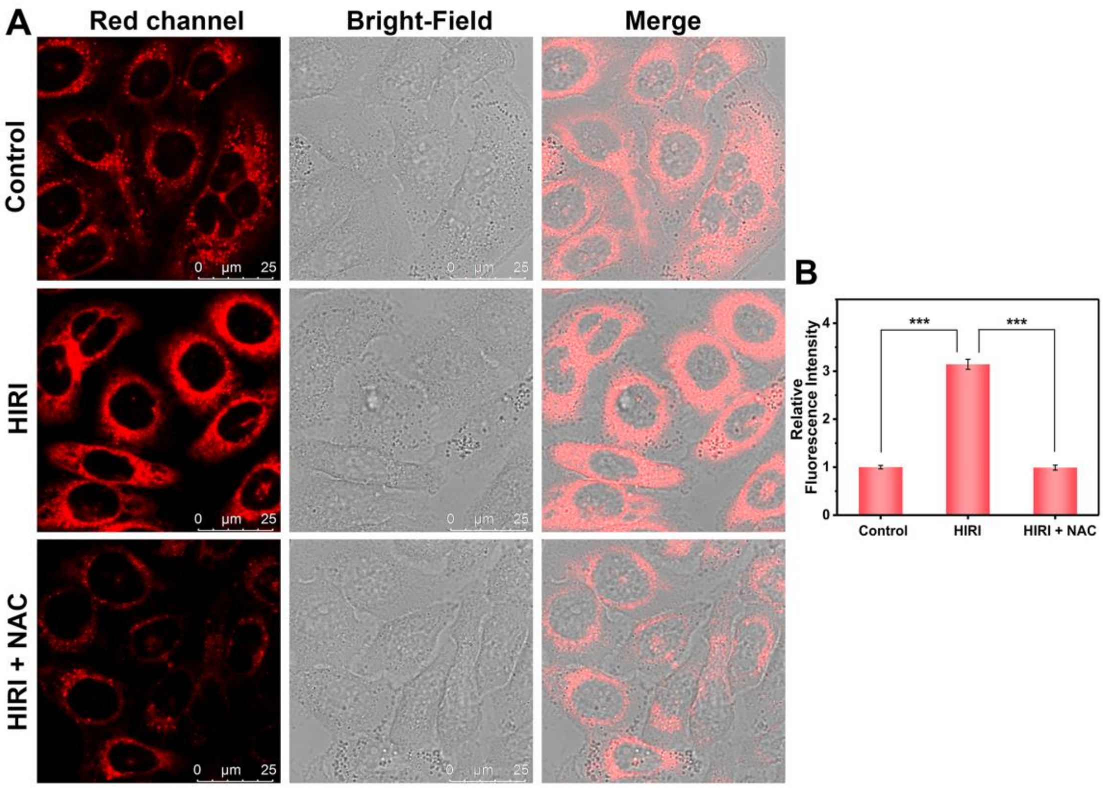

Figure S13. Confocal fluorescence imaging of lysosomal viscosity in hepatocytes under various treatments. (A) Control hepatocytes, HIRI hepatocytes and HIRI + NAC hepatocytes were incubated 20 μM NP-V for the detection of lysosomal viscosity. Ex= 633 nm, collected 640−820 nm. (B) Relative fluorescence outputs of the three groups. The red fluorescence of control group was defined as 1. The data are expressed as the mean ± SD. ***$P < 0.001$. Concordant results were obtained from three independent experiments.

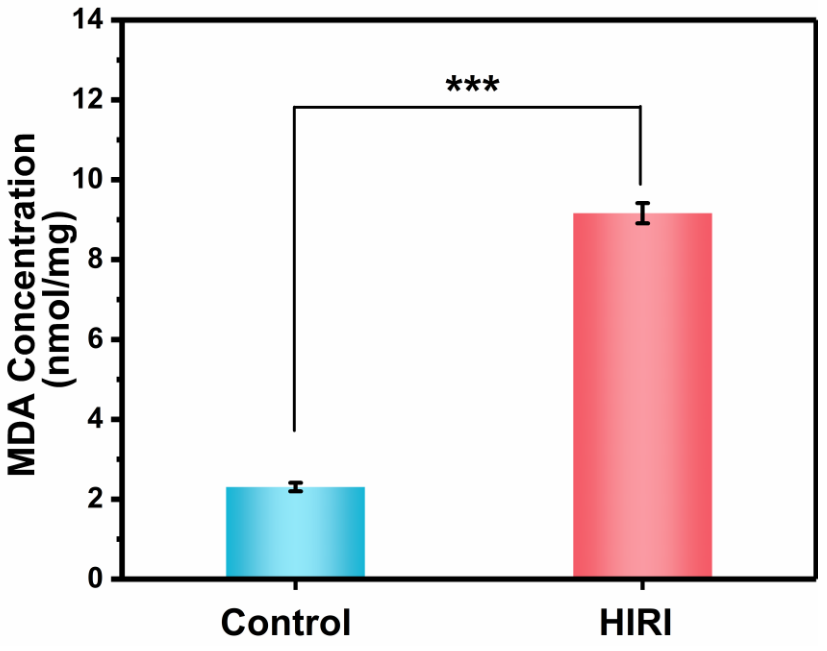

Figure S14. The lysosomal MDA concentrations in the control and HIRI groups. The data are expressed as the mean ± SD. ***P < 0.001.

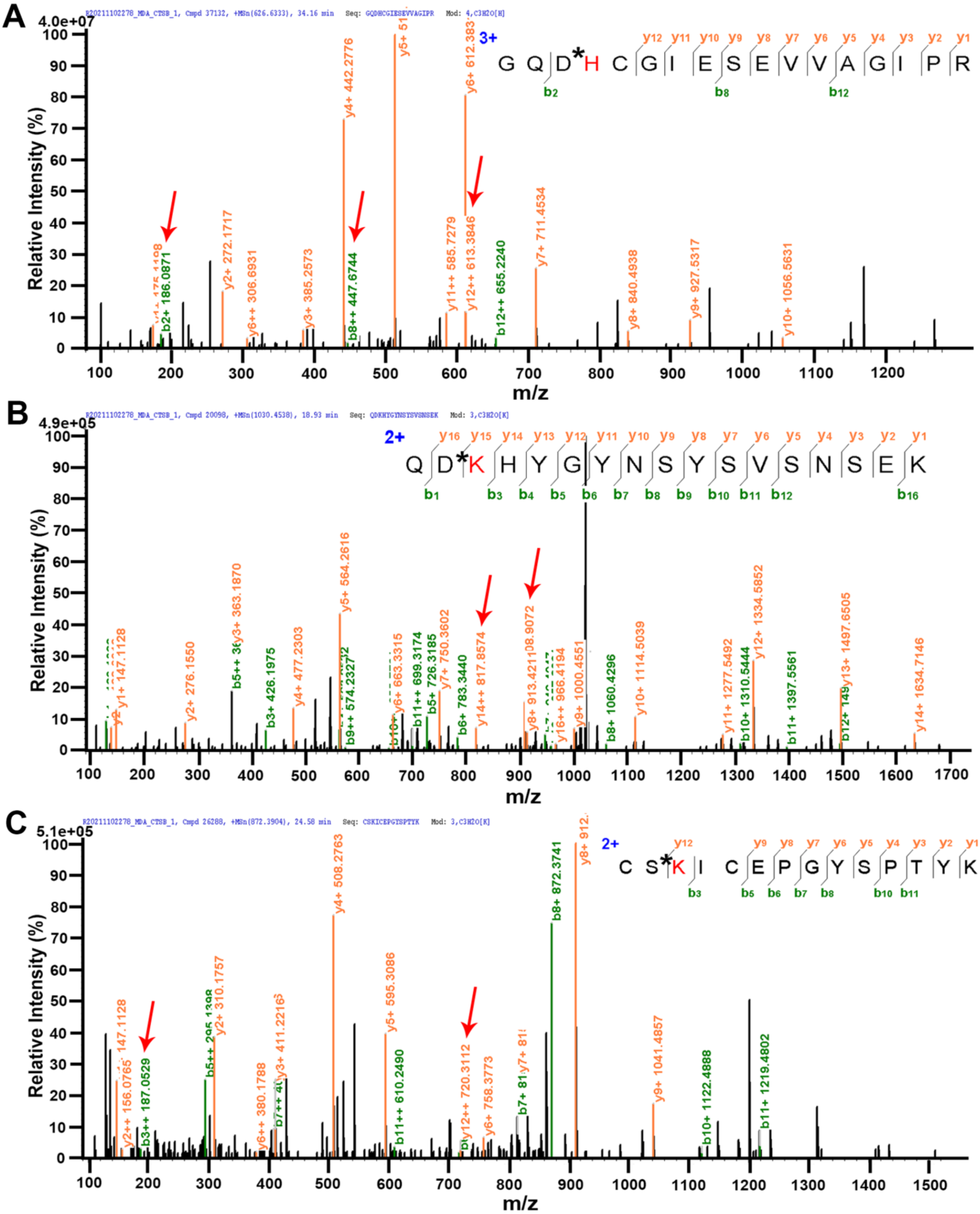
A
4.0e+07
3+
G Q D*H C G I E S E V V A G I P R
y12 y11 y10 y9 y8 y7 y6 y5 y4 y3 y2 y1
b2 b8 b12
y4+ 442.2776
y6+ 612.383
y2+ 272.1717
y6++ 306.6931
y3+ 385.2573
b2+ 186.0871
b8++ 447.6744
y11++ 585.7279
y12++ 613.3846
b12++ 655.2240
y7+ 711.4534
y8+ 840.4938
y9+ 927.5317
y10+ 1056.5631
Relative Intensity (%)
m/z
B
4.9e+05
2+
Q D*K H Y G Y N S Y S V S N S E K
y16 y15 y14 y13 y12 y11 y10 y9 y8 y7 y6 y5 y4 y3 y2 y1
b1 b3 b4 b5 b6 b7 b8 b9 b10 b11 b12 b16
y1+ 147.1128
y2+ 276.1550
y3+ 363.1870
b3+ 426.1975
y4+ 477.2303
y5+ 564.2616
b9++ 574.2327
y6+ 663.3315
b11++ 699.3174
b5+ 726.3185
y7+ 750.3602
b6+ 783.3440
y14++ 817.8574
y16++ 966.4194
y9+ 1000.4551
b8+ 1060.4296
y10+ 1114.5039
y11+ 1277.5492
b10+ 1310.5444
y12+ 1334.5852
b11+ 1397.5561
y13+ 1497.6505
y14+ 1634.7146
Relative Intensity (%)
m/z
C
5.1e+05
2+
C S*K I C E P G Y S P T Y K
y12 y9 y8 y7 y6 y5 y4 y3 y2 y1
b3 b5 b6 b7 b8 b10 b11
y2++ 156.0765
b3++ 187.0529
y2+ 310.1757
y6++ 380.1788
y3+ 411.2216
y4+ 508.2763
y5+ 595.3086
b11++ 610.2490
y12++ 720.3112
y6+ 758.3773
b8+ 872.3741
y9+ 1041.4857
b10+ 1122.4888
b11+ 1219.4802
Relative Intensity (%)
m/z

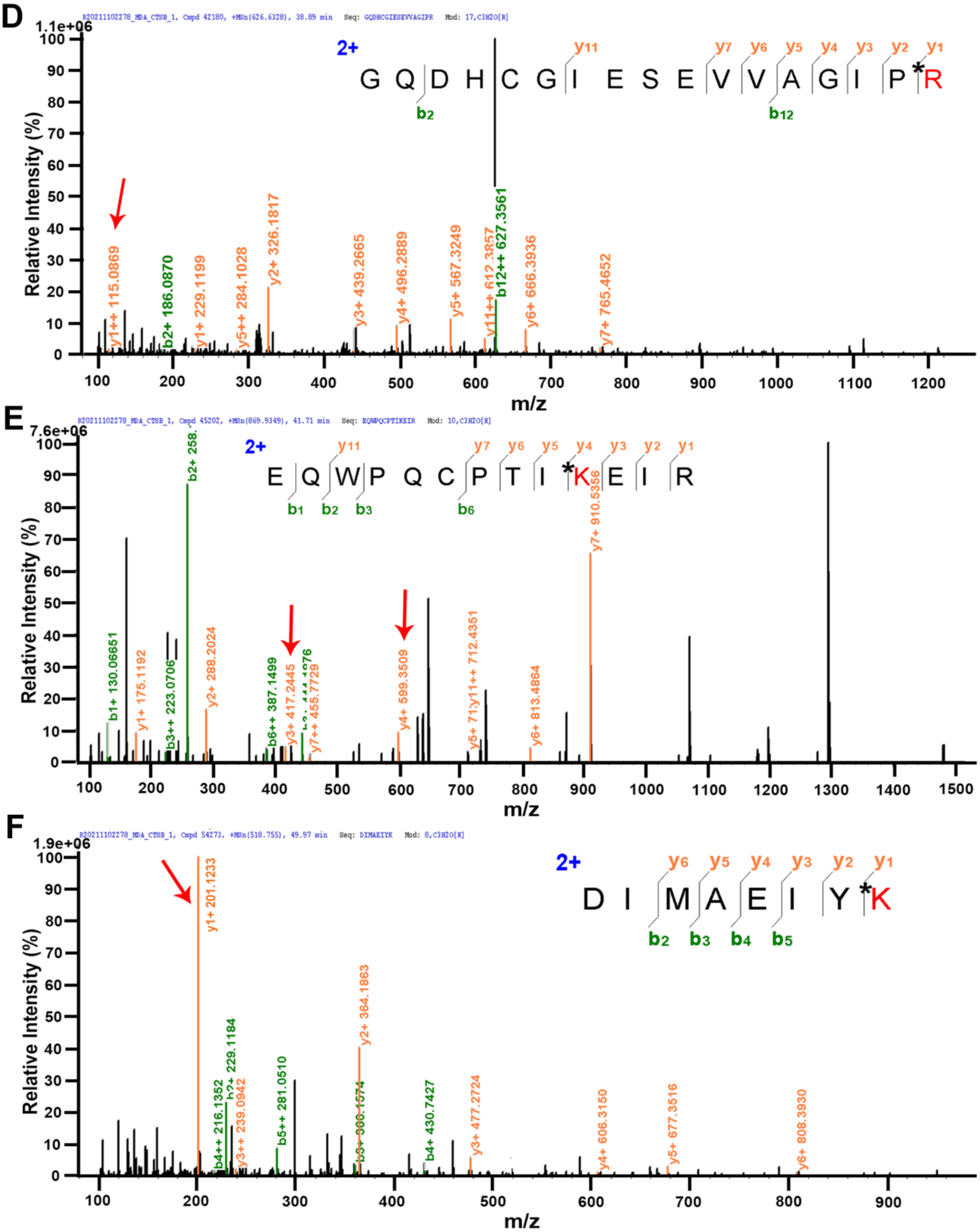
D
R20211102278_MDA_CTSB_1, Cmpd 42180, +MSn(626.6328), 38.89 min Seq: GQDHCGIESEVVAGIPR Mod: 17,C3H2O[R]
1.1e+06
2+
G Q D H C G I E S E V V A G I P *R
y11 y7 y6 y5 y4 y3 y2 y1
b2 b12
y1++ 115.0869
b2+ 186.0870
y1+ 229.1199
y5++ 284.1028
y2+ 326.1817
y3+ 439.2665
y4+ 496.2889
y5+ 567.3249
y11++ 612.3857
b12++ 627.3561
y6+ 666.3936
y7+ 765.4652
Relative Intensity (%)
m/z
E
R20211102278_MDA_CTSB_1, Cmpd 45202, +MSn(869.9349), 41.71 min Seq: EQWPQCPTIKEIR Mod: 10,C3H2O[K]
7.6e+06
2+
E Q W P Q C P T I *K E I R
y11 y7 y6 y5 y4 y3 y2 y1
b1 b2 b3 b6
b1+ 130.06651
y1+ 175.1192
b3++ 223.0706
b2+ 258.
y2+ 288.2024
b6++ 387.1499
y3+ 417.2445
y7++ 455.7729
y4+ 599.3509
y11++ 712.4351
y6+ 813.4864
y7+ 910.5356
Relative Intensity (%)
m/z
F
R20211102278_MDA_CTSB_1, Cmpd 54273, +MSn(518.755), 49.97 min Seq: DIMAEIYK Mod: 8,C3H2O[K]
1.9e+06
2+
D I M A E I Y *K
y6 y5 y4 y3 y2 y1
b2 b3 b4 b5
y1+ 201.1233
b4++ 216.1352
b2+ 229.1184
y3++ 239.0942
b5++ 281.0510
y2+ 364.1863
b4+ 430.7427
y3+ 477.2724
y4+ 606.3150
y5+ 677.3516
y6+ 808.3930
Relative Intensity (%)
m/z

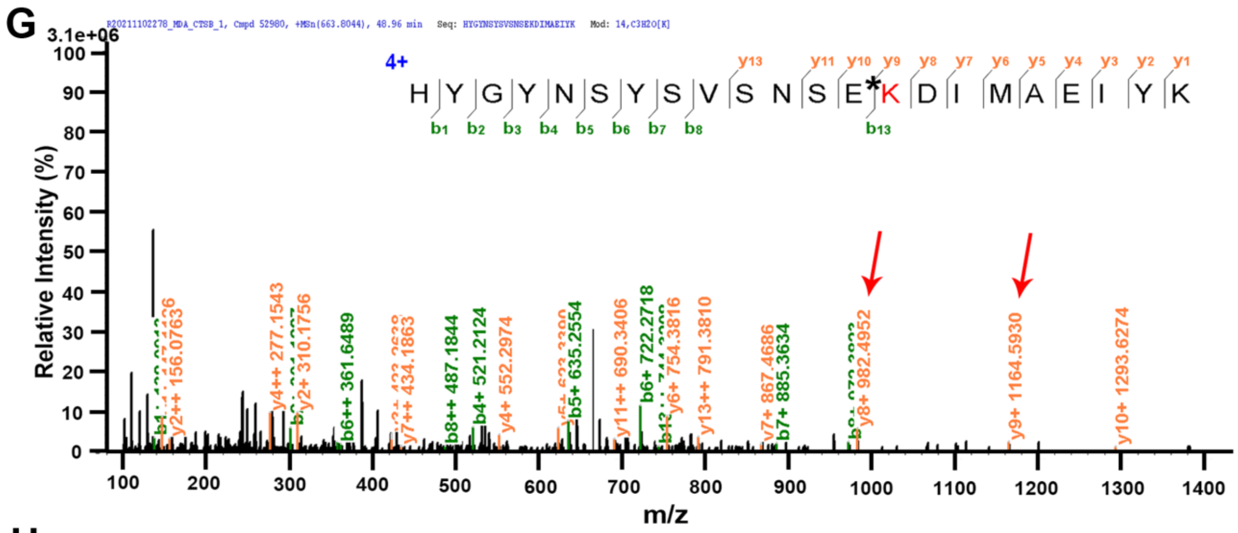

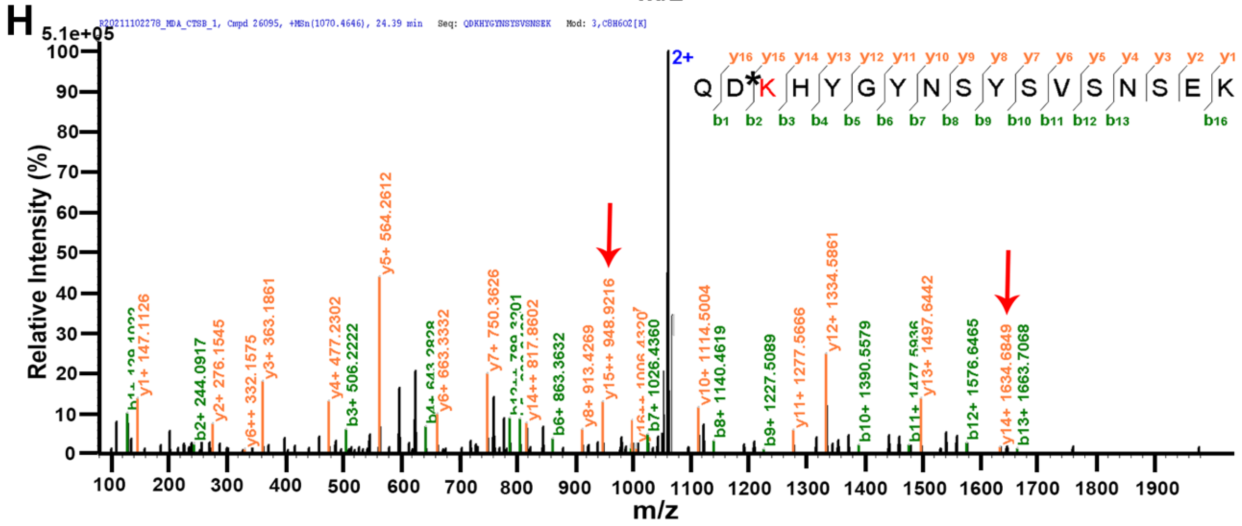

Figure S15. Proteomic analysis of the reaction of cathepsin B with MDA through LC-MS/MS. MDA Schiff base adducts (+54 Da) on H318 (A), K223 (B), K209 (C), R331 (D), K97 (E), K245 (F), K237 (G). Dihydropyridine (DHP) type MDA adducts (+134 Da) on K223 (H). Amino acids labeled with * and red color denote modification by MDA. The relevant peaks due to amino acids modified by MDA (+54 Da or +134 Da) were highlighted by red arrows. Note: In Figure S15B, the y_{15}^{++} peak which is highlighted by the second red arrow is 908.9072.

	MDA 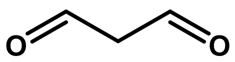
Lys	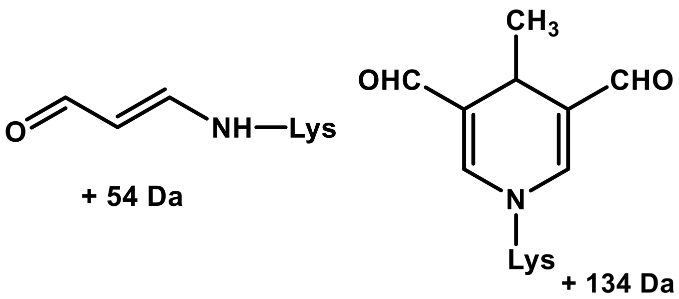
His	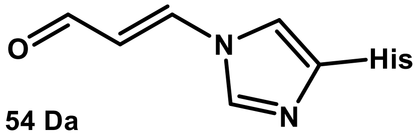
Arg	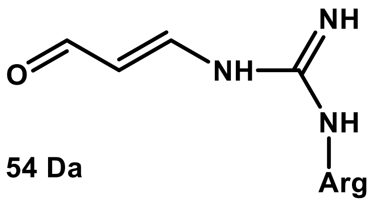
Asn	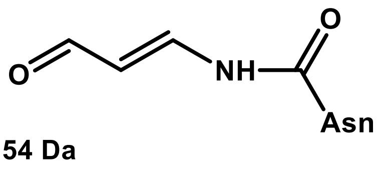

Table S2. The predicted structures of MDA-modified adducts with amino acid residues (lysine, histidine, arginine and asparagine) in cathepsin B.

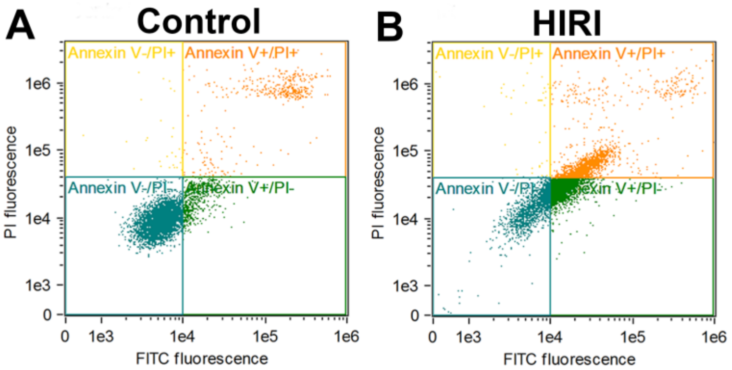

Figure S16. Flow cytometric analysis of control (A) and HIRI (B) hepatocytes.

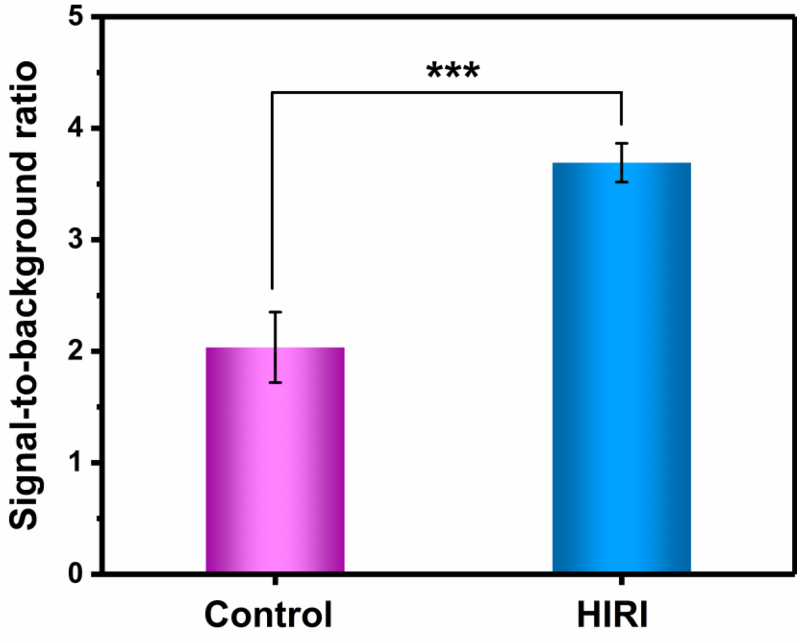

Figure S17. Calculated signal-to-background ratio (SBR) in mice. The SBR was defined as signals from liver site relative to skin. The data are expressed as the mean ± SD. ***P < 0.001. Four mice in each group.

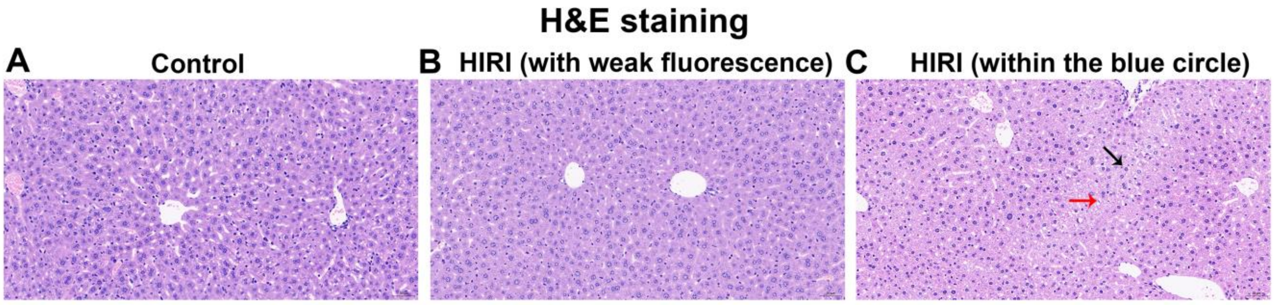

Figure S18. H&E staining for the liver tissues within the blue circle (C) and with weak fluorescence (B) of HIRI group as well as some control group liver tissues (A). (A), (B) Hepatic lobules are well demarcated and regularly arranged. No obvious expansion or extrusion of hepatic sinuses were observed. There was no obvious abnormality in the portal area between adjacent hepatic lobules. No obvious inflammatory changes were observed. (C) The resection tissues within the blue circle exhibited a large amount of granular degeneration of hepatocytes, cell swelling, loose and light staining cytoplasm with fine granular shape (black arrow), and steatosis with small round vacuoles in the cytoplasm (red arrow).

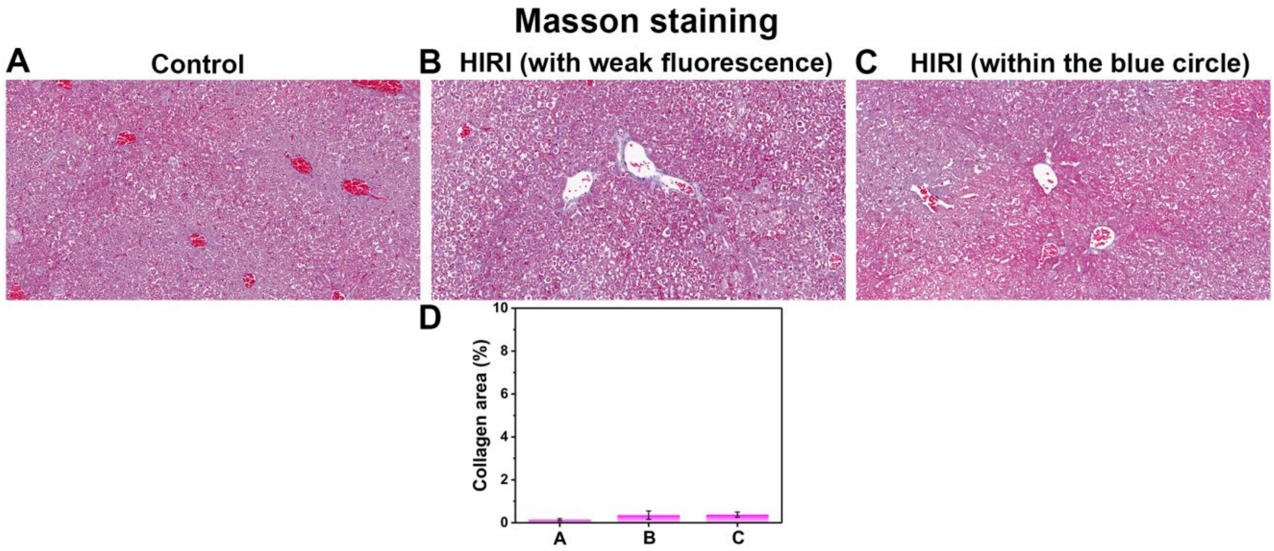

Figure S19. Masson staining for the liver tissues within the blue circle (C) and with weak fluorescence (B) of HIRI group as well as some control group liver tissues (A). (D) Percentage of collagen area (%).

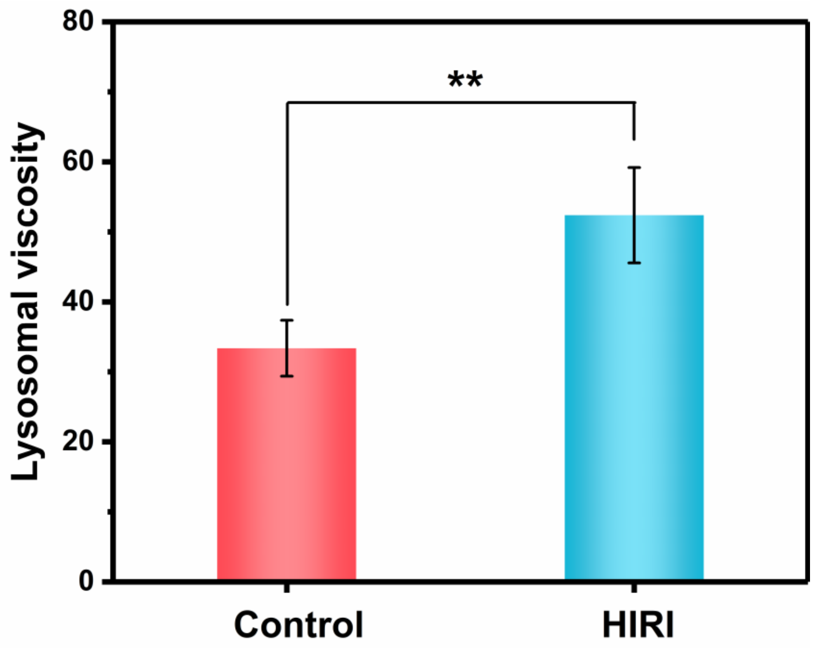

Figure S20. The lysosomal viscosity of the livers from control and HIRI group mice using an NDJ-8S rotational viscometer. The data are expressed as the mean ± SD. **P < 0.01. Four mice in each group.

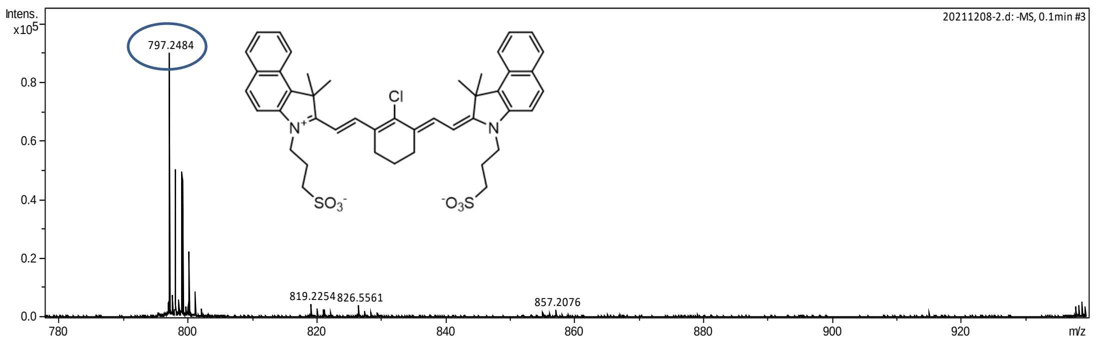

Figure S21. HRMS of NP-V.

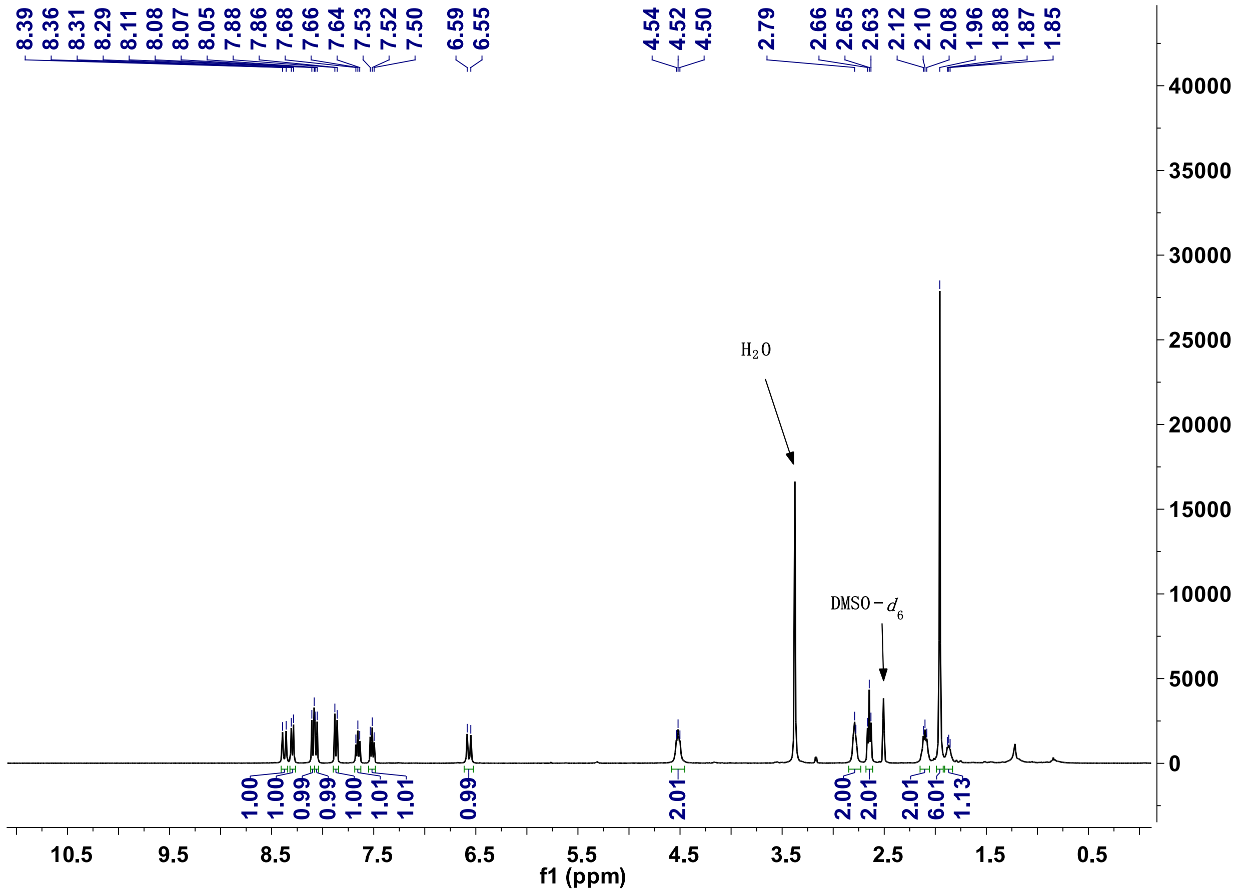

Figure S22. ^{1}H NMR (400 MHz, DMSO-d_6) of NP-V. The relevant peaks arising from the solvent (DMSO-d_6) are highlighted by black arrows.

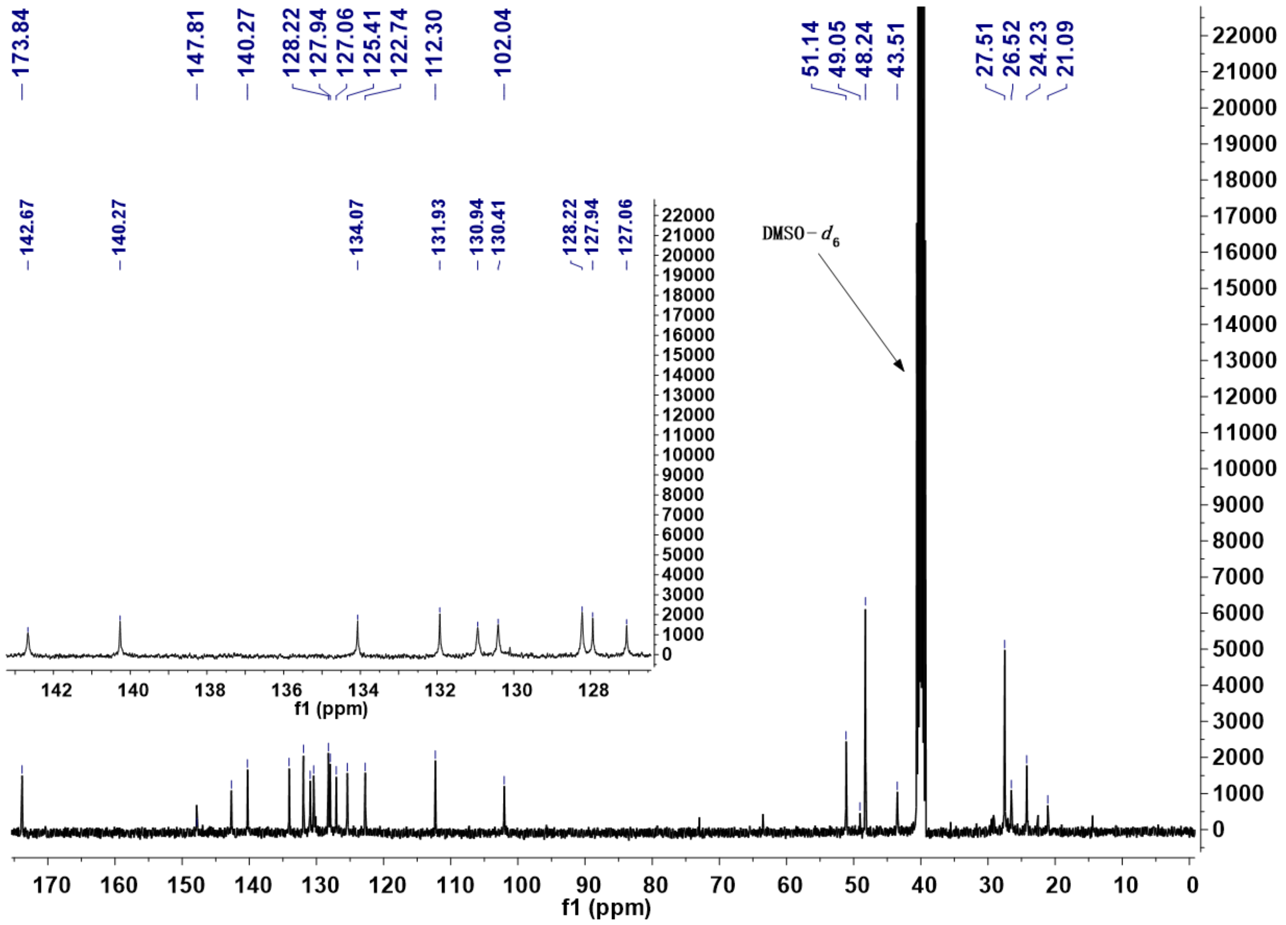

Figure S23. ^{13}C NMR (100 MHz, DMSO-*d*$_6$) of NP-V. The relevant peaks arising from the solvent (DMSO-*d*$_6$) are highlighted by black arrows.

References

(1) Tsuruta, Y.; Date, Y.; Tonogaito, H.; Sugihara, N.; Furuno, K.; Kohashi, K. Determination of malondialdehyde by high-performance liquid chromatography using 4-(2-phthalimidyl)benzohydrazide as a pre-column fluorescent labelling reagent. *Analyst* **1994**, *119*, 1047-1050.

(2) Kwon, T.-W.; Watts, B. M. Determination of malonaldehyde by ultraviolet spectrophotometrya. *J. Food Sci.* **1963**, *28*, 627-630.

(3) Dou, K.; Huang, W.; Xiang, Y.; Li, S.; Liu, Z. Design of activatable NIR-II molecular probe for in vivo elucidation of disease-related viscosity variations. *Anal. Chem.* **2020**, *92*, 4177-4181.

(4) Antaris, A. L.; Chen, H.; Diao, S.; Ma, Z.; Zhang, Z.; Zhu, S.; Wang, J.; Lozano, A. X.; Fan, Q.; Chew, L.; Zhu, M.; Cheng, K.; Hong, X.; Dai, H.; Cheng, Z. A high quantum yield molecule-protein complex fluorophore for near-infrared II imaging. *Nat. Commun.* **2017**, *8*, 15269.

(5) Chen, Q.; Wang, C.; Zhan, Z.; He, W.; Cheng, Z.; Li, Y.; Liu, Z. Near-infrared dye bound albumin with separated imaging and therapy wavelength channels for imaging-guided photothermal therapy. *Biomaterials* **2014**, *35*, 8206-8214.

(6) Zhao, J.; Chen, J.; Zhu, H.; Xiong, Y. L. Mass spectrometric evidence of malonaldehyde and 4-hydroxynonenal adductions to radical-scavenging soy peptides. *J. Agric. Food Chem.* **2012**, *60*, 9727-9736.
